# RGS5 Determines Neutrophil Migration in the Acute Inflammatory Phase of Bleomycin-Induced Lung Injury

**DOI:** 10.3390/ijms22179342

**Published:** 2021-08-28

**Authors:** Neha Sharma, Chandran Nagaraj, Bence M. Nagy, Leigh M. Marsh, Natalie Bordag, Diana Zabini, Malgorzata Wygrecka, Walter Klepetko, Elisabeth Gschwandtner, Guillem Genové, Akos Heinemann, E Kenneth Weir, Grazyna Kwapiszewska, Horst Olschewski, Andrea Olschewski

**Affiliations:** 1 Ludwig Boltzmann Institute for Lung Vascular Research, 8010 Graz, Austria; neha.sharma@stud.medunigraz.at (N.S.); Nagaraj.Chandran@lvr.lbg.ac.at (C.N.); Bence.Nagy@lvr.lbg.ac.at (B.M.N.); leigh.marsh@lvr.lbg.ac.at (L.M.M.); natalie.bordag@lvr.lbg.ac.at (N.B.); d.zabini@medunigraz.at (D.Z.); grazyna.kwapiszewska@lvr.lbg.ac.at (G.K.); horst.olschewski@medunigraz.at (H.O.); 2 Experimental Anaesthesiology, Department of Anaesthesiology and Intensive Care Medicine, Medical University of Graz, 8036 Graz, Austria; 3 Department of Dermatology and Venereology, Medical University of Graz, 8036 Graz, Austria; 4 Otto Loewi Research Center, Medical University of Graz, 8010 Graz, Austria; 5 Department of Biochemistry, Universities of Giessen and Marburg Lung Center, Justus Liebig University of Giessen, Friedrichstrasse 24, 35392 Giessen, Germany; malgorzata.wygrecka@innere.med.uni-giessen.de; 6 Department of Thoracic Surgery, Medical University of Vienna, 1090 Vienna, Austria; walter.klepetko@meduniwien.ac.at (W.K.); elisabeth.gschwandtner@meduniwien.ac.at (E.G.); 7 Integrated CardioMetabolic Centre (ICMC), Department of Medicine, Karolinska Institute, 171 77 Huddinge, Sweden; guillem.genove@ki.se; 8 Otto Loewi Research Center for Vascular Biology, Immunology and Inflammation, Division of Pharmacology, Medical University of Graz, 8010 Graz, Austria; akos.heinemann@medunigraz.at; 9 Department of Medicine, University of Minnesota, Minneapolis, MN 55455, USA; weirx002@umn.edu; 10 Department of Internal Medicine, Division of Pulmonology, Medical University of Graz, 8036 Graz, Austria

**Keywords:** regulator of G protein signaling 5, neutrophilic inflammation, chemotaxis, ERK, interstitial lung diseases

## Abstract

The regulator of G protein signaling (RGS) represents a widespread system of controllers of cellular responses. The activities of the R4 subfamily of RGSs have been elucidated in allergic pulmonary diseases. However, the R4 signaling in other inflammatory lung diseases, with a strong cellular immune response, remained unexplored. Thus, our study aimed to discern the functional relevance of the R4 family member, RGS5, as a potential modulating element in this context. Gene profiling of the R4 subfamily showed increased RGS5 expression in human fibrosing lung disease samples. In line with this, RGS5 was markedly increased in murine lungs following bleomycin injury. RGS knock-out mice (RGS-/-) had preserved lung function while control mice showed significant combined ventilatory disorders three days after bleomycin application as compared to untreated control mice. Loss of RGS5 was associated with a significantly reduced neutrophil influx and tissue myeloperoxidase expression. In the LPS lung injury model, RGS5-/- mice also failed to recruit neutrophils into the lung, which was accompanied by reduced tissue myeloperoxidase levels after 24 h. Our in-vitro assays showed impaired migration of RGS5-/- neutrophils towards chemokines despite preserved Ca^2+^ signaling. ERK dephosphorylation might play a role in reduced neutrophil migration in our model. As a conclusion, loss of RGS5 preserves lung function and attenuates hyperinflammation in the acute phase of bleomycin-induced pulmonary fibrosis and LPS-induced lung injury. Targeting RGS5 might alleviate the severity of exacerbations in interstitial lung diseases.

## 1. Introduction

G protein-coupled receptor (GPCR) signaling is essential for the pathogenesis of inflammatory diseases and represents one of the most frequently targeted receptor pathways in modern pharmacotherapy. The downstream consequences of GPCR signaling are determined by different regulatory proteins including regulators of G protein signaling (RGS) [1]. RGS proteins negatively modulate the amplitude and duration of the signal. Biochemically, RGS catalyze GTPase activity of the G protein Gα-subunit which rapidly facilitates hydrolysis of GTP to GDP and stabilizes the Gα and Gβγ G protein heterotrimeric complex [2]. RGS are abundantly expressed in the lung and in inflammatory cells, where they may regulate inflammatory responses to immune challenge [3]. Previous studies suggested that RGS2, RGS4 and RGS5 were protective against airway-hyperresponsiveness with modulated airway or bronchial contractility [4,5,6], while RGS2 was protective against bleomycin-induced lung fibrosis [7]. These specific RGS proteins are members of the R4 subfamily. They are the smallest RGS proteins in size. The highly homologous primary structure contains only short peptide sequences flanking the RGS box, with the exception of RGS3 [8]. As human diseases directly attributable to dysfunction of a specific R4 RGS protein are not known, it is challenging to delineate the pathways regulated by each of these proteins. Some R4 activities in allergic pulmonary diseases have been elucidated, but it’s signaling involved in other inflammatory lung diseases with a strong cellular immune response followed by fibrosis has not been investigated. 

Interstitial lung diseases (ILDs) are important causes of morbidity and mortality worldwide and despite great advances in specific therapies, the management of the diseases is often complicated [9,10] due to frequent and life-threatening exacerbations that represent an unsolved clinical problem. Overexuberant neutrophil influx, cytokine release, epithelial damage, remodeling and fibrosis are central features of the diseases, particularly during flare-ups. The inflammation may be present in the airways and/or in the parenchyma of the lung and consists of acute and chronic components with much overlap between disease phenotypes. Therefore, we decided, rather than focusing on a single disease, to investigate a potential control element of the inflammatory process. Our systematic evaluation of RGS proteins of the R4 subfamily showed striking upregulation of RGS5 in human fibrosing lung disease samples. Thus, we hypothesized that RGS5 might play an important role in disease development and applied two independent animal models resembling the different stages of acute interstitial inflammation and fibrosing lung disease. Using RGS5 knockout animals, we found a selective effect of RGS5 on the neutrophilic inflammatory response in the early phase of acute respiratory distress syndrome (ARDS). 

## 2. Results

### 2.1. Fibrosing Interstitial Lung Diseases Are Associated with Changes in R4 Expression

**Figure 1:**
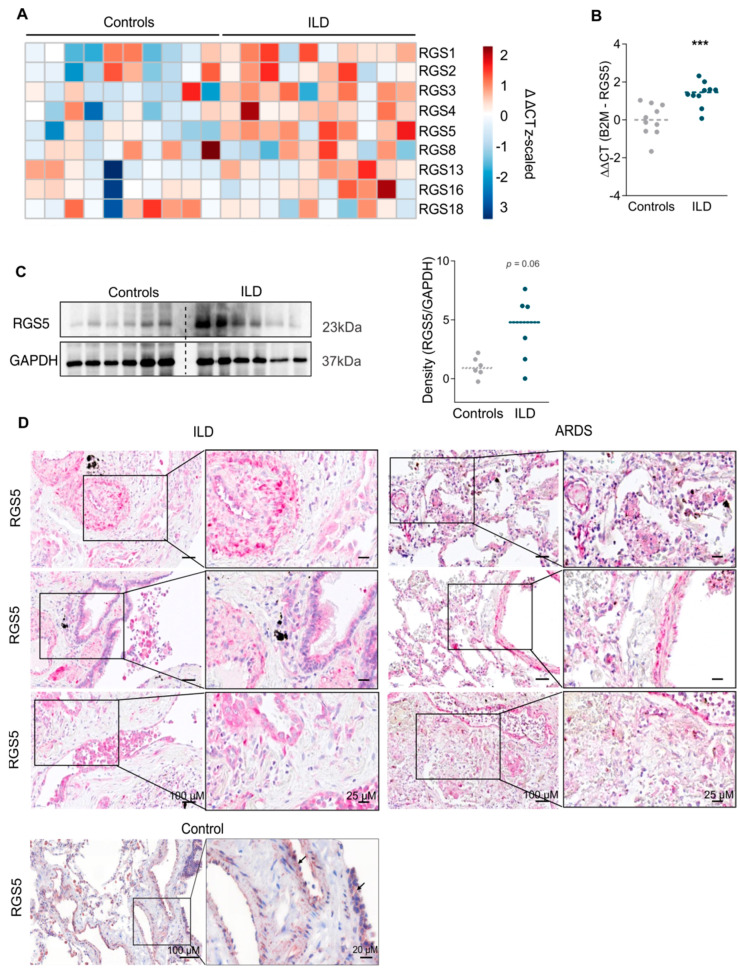
Increased RGS5 expression in interstitial lung disease (ILD) and acute respiratory distress syndrome (ARDS) in tissue from patients undergoing lung transplantation. (**A**) Heat map depicting the expression (RT-qPCR) of the RGS subfamily members (RGS1, 2, 3, 4, 5, 8, 13, 16, 18) in lungs from healthy controls (*n* = 10 different patients) and ILD patients (*n* = 10). (**B**) RGS5 mRNA expression in the lungs of healthy controls (*n* = 10) and ILD patients (*n* = 10). ΔΔCT values are calculated by ΔCT (target gene)—ΔCT (averaged controls). Data are presented as scatter dot plot and analyzed using unpaired two-tailed *t*-test, *** *p* < 0.001. (**C**) RGS5 protein levels compared via immunoblotting in lungs from controls (*n* = 6) and ILD patients (*n* = 6). GAPDH served as a loading control. (**D**) Representative cross-sections (*n* = 3) of ILD, ARDS (dark pink) and controls (brown) lung tissues showing RGS5 expression by immunohistochemical (IHC) staining; scale bar: 100, 25 and 20 µm.

The gene expression of the R4 subfamily members showed up-regulated RGS1, RGS3, RGS4 and RGS5 in fibrosing ILD compared to healthy controls (healthy controls = 10, ILD = 10) (Figure 1A,B). The expression of RGS2, RGS8, RGS13, RGS16 and RGS18 was not changed. RGS5 was also markedly increased on the protein level in ILD lungs (Figure 1C). The clinical characteristics of the study population and the use of the samples are given in Supplementary Tables S1 and S2. Immunohistochemical (IHC) staining showed that RGS5 is present in airway and vascular structural cells, as well as in inflammatory cells in controls and ILD, as well as in samples obtained from patients with acute respiratory distress (ARDS) (Figure 1D and Figure S1). Subsequently, we focused on the inflammatory cells in our study.

**Figure 2:**
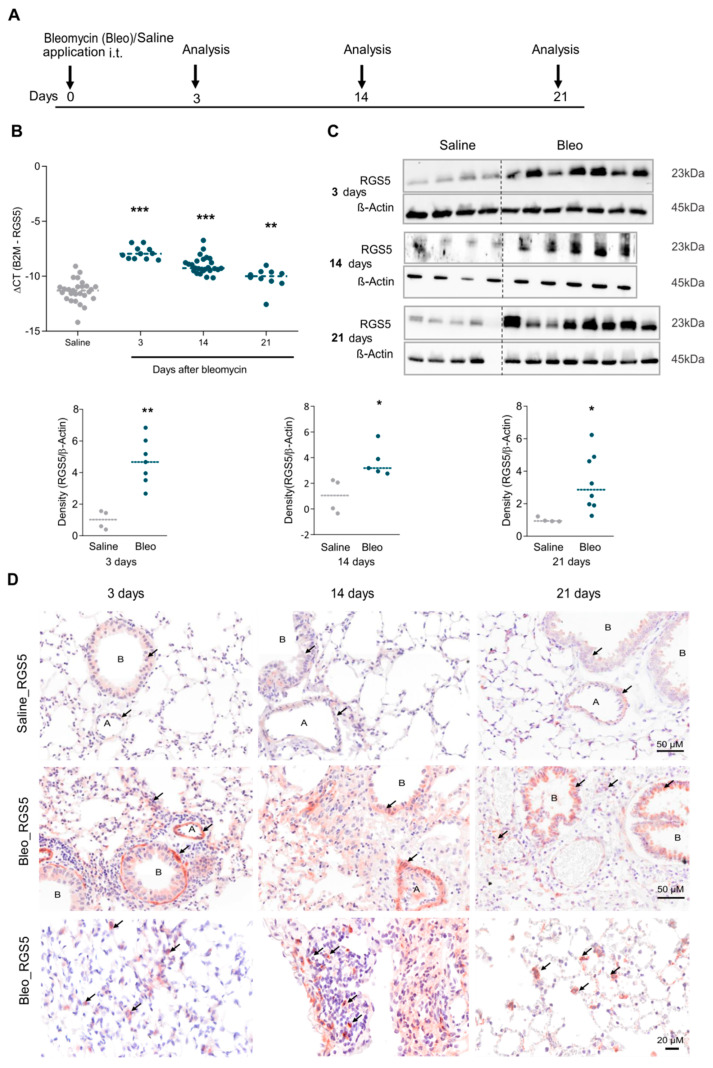
Regulation of RGS5 expression in mouse lungs after bleomycin treatment. (**A**) Schematic overview of the bleomycin challenge. (**B**) mRNA expression of RGS5 in the lung after bleomycin injury. ΔCT values, calculated by ΔCT = CT (reference gene)—CT (target gene) (minimum *n* = 10 different mice). Data are presented as scatter dot plots and analyzed using one-way ANOVA with Bonferroni’s multiple comparison test, ** *p* < 0.01, *** *p* < 0.001. (**C**) RGS5 protein expression levels are shown via immunoblotting. β-actin served as a loading control as shown in a representative image (*n* = 4 to 8 mice). Data are presented as scatter dot plots and analyzed using unpaired two-tailed *t*-test, * *p* < 0.05; ** *p* < 0.01. (**D**) Representative cross-sections (*n* = 3) of the lung tissue obtained from saline- (upper panel) and bleomycin- (middle and lower panel) treated animals. Selection of positive RGS5 staining (brown by IHC staining) in structural and inflammatory cells are indicated by arrows; scale bar: 20 or 50 µm as indicated. A = pulmonary arteries, B = bronchioles.

Next, we utilized the bleomycin mouse model, a well-established animal model for both acute inflammatory lung injury and lung fibrosis and investigated the longitudinal RGS5 expression. Mice were challenged intratracheally (i.t) with bleomycin sulfate or saline (control) and harvested on days 3, 14, and 21 to assess inflammation and fibrosis (Figure 2A). mRNA levels and protein expression of RGS5 at 3, 14 and 21 days in the lung homogenates were consistently elevated (Figure 2B,C). The presence of RGS5 was assessed by IHC staining on lung sections, showing RGS5 in the airway and vascular structural cells and in inflammatory cells, with a more marked appearance in bleomycin-treated animals compared to saline-treated mice (Figure 2D and Figure S1). 

### 2.2. RGS5 Deficiency Protects Mice from Bleomycin-Induced Acute Inflammatory Response in the Lung

**Figure 3:**
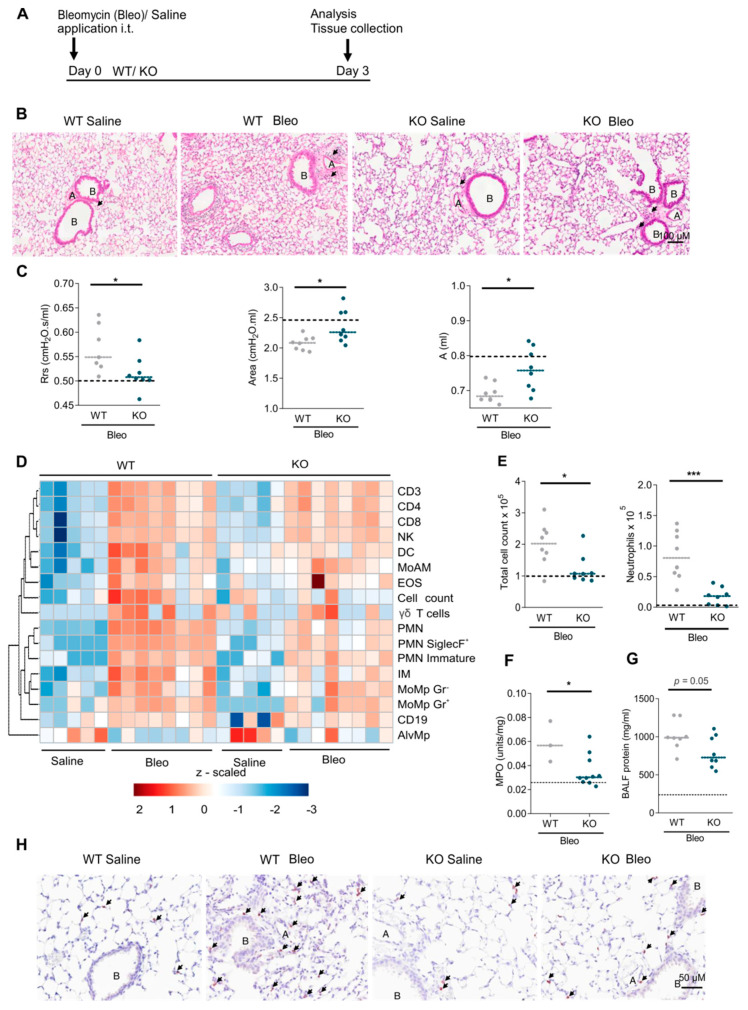
Loss of RGS5 protects the lungs in the early phase from bleomycin-induced lung injury. (**A**) Schematic representation of bleomycin treatment. (**B**) Hematoxylin and Eosin (H&E) staining, arrows indicating the inflammatory cells; scale bar: 100 µM. A = pulmonary arteries, B = bronchioles. (**C**) Lung function measurements showing the airway resistance (Rrs), the area enclosed under P-V loop and estimation of inspiratory capacity (**A**). (**D**) Heatmap presenting flow-cytometry of inflammatory cell populations in BALF 3 days after saline and bleomycin-challenge (log_10_(x + 1) transformed, z-scaled per cell type) shown with average distance sorted dendrograms from hierarchical clustering analysis. (**E**) Total cell count and neutrophil numbers in BALF. (**F**) Myeloperoxidase (MPO) activity in the lung tissue. (**G**) Bronchoalveolar lavage fluid (BALF) protein measurements using BCA assay. (**H**) Anti-Ly6G IHC staining indicates neutrophils in the lung tissue, arrows indicating neutrophils; scale bar: 50 µM. Dotted lines represent mean of WT and RGS5-/- saline-treated pooled together, as no differences were observed. The dashed lines in (**C**,**E**–**G**) show the mean values in control animals. Data are presented as scatter dot plot (*n* = 3–10 different mice) and analyzed using unpaired two-tailed *t*-test, * *p* < 0.05; *** *p* < 0.001.

To investigate the role of RGS5 in vivo we examined the acute inflammatory response phase of pulmonary fibrosis utilizing RGS5-/- and WT at age 12–16 wk (equivalent to adult age in humans) (Figure 3A). At baseline, RGS5-/- showed no differences in lung function and pulmonary hemodynamic parameters as compared to WT (Supplementary Figure S2A,B). On day 3 after bleomycin treatment, we found significant differences between RGS5-/- and WT. Histological evaluation revealed an elevated number of immune cells in the lungs of bleomycin treated WT and RGS-/- mice, accumulating around bronchioles and pulmonary vessels (Figure 3B). Importantly, in the lung function testing, RGS-/-, as compared to WT, showed no increase in airway resistance (Rrs) and no reduction in lung compliance (area under PV loop) and maintained their normal inspiratory capacity (A) (Figure 3C and Figure S3A). Heatmap analysis of specific myeloid and lymphoid immune cell subpopulations in BALF and lung tissue showed that RGS5-/- had a significant attenuation in the cellular response, predominantly made up of neutrophils in BALF (Figure 3D,E and Figure S3B). Furthermore, myeloperoxidase (MPO) enzymatic activity in the lung tissue was significantly lowered in RGS5-/- (Figure 3F). The BALF protein response, representing alveolar leakage in the lungs, was blunted in RGS5-/- on day 3 after bleomycin challenge (Figure 3G). Ly6G staining on the lung sections confirmed reduced neutrophil recruitment in RGS5-/- (Figure 3H). Changes in additional inflammatory gene expressions are sown in Supplementary Figure S7.

### 2.3. RGS5 Is Essential for Neutrophil Recruitment in the Lung upon LPS Injury

**Figure 4:**
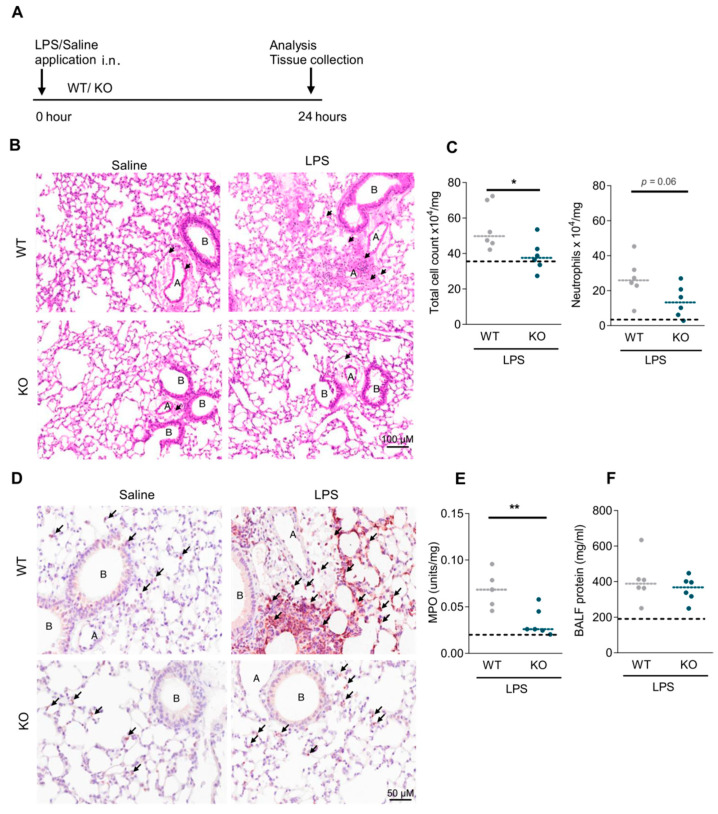
Loss of RGS5 impairs neutrophil recruitment in LPS-induced lung injury. (**A**) Schematic overview of the LPS treatment. (**B**) H&E staining, arrows indicating the inflammatory cells; scale bar: 100 µM. A = pulmonary arteries, B = bronchioles. (**C**) Total cell and neutrophil numbers from lung tissue. (**D**) Anti-Ly6G IHC staining for neutrophils on lung tissue cross-sections, arrows indicating neutrophils; scale bar: 50 µM. A = pulmonary arteries, B = bronchioles. (**E**) MPO activity in the lung tissue (**F**) BALF protein measurements using BCA assay. Dotted lines represent means of WT and RGS5-/- saline-treated pooled together, as no differences were observed. The dashed lines in C, E, and F show the mean values in control animals. Data are presented as scatter dot plots (*n* = 4–6 mice) and analyzed using unpaired two-tailed *t*-test, * *p* < 0.05; ** *p* < 0.01.

To conclusively evaluate the impact of RGS5 deficiency in acute inflammatory lung disease, we additionally utilized a second animal model, the lipopolysaccharide (LPS)-induced acute lung injury model (Figure 4A). RGS5-/- showed a significantly attenuated response in comparison to WT which showed a massive infiltration of inflammatory cells in the lung tissue by 24 h after LPS (Figure 4B). In detail; in RGS5-/- the total cell count was significantly reduced, mainly due to reduced neutrophil numbers in the lung tissue (Figure 4C). The reduced neutrophil influx was further confirmed by the neutrophil-specific anti-Ly6G IHC staining on lung tissue cross-sections (Figure 4D). In line with this, MPO activity in the lungs was significantly lower in RGS5-/- upon LPS challenge, (Figure 4E), while BALF protein (Figure 4F) and total cell count, neutrophil cell count, CXCL1, and CXCL2 were not significantly changed by RGS5-/- compared with WT (Supplementary Figure S4A–D). Changes in additional inflammatory gene expressions are sown in Supplementary Figure S7. 

### 2.4. Neutrophils Obtained from RGS5 Deficient Mice Show Markedly Impaired Chemotactic Motility 

Our data indicated that RGS5 is essential for recruitment of neutrophils in the lungs during the acute inflammatory phase. To explore the molecular mechanisms, we investigated the chemokine levels in the lung, the homeostatic neutrophil localization and the migratory behavior of the neutrophils obtained from WT and RGS5-/- in vitro. 

**Figure 5:**
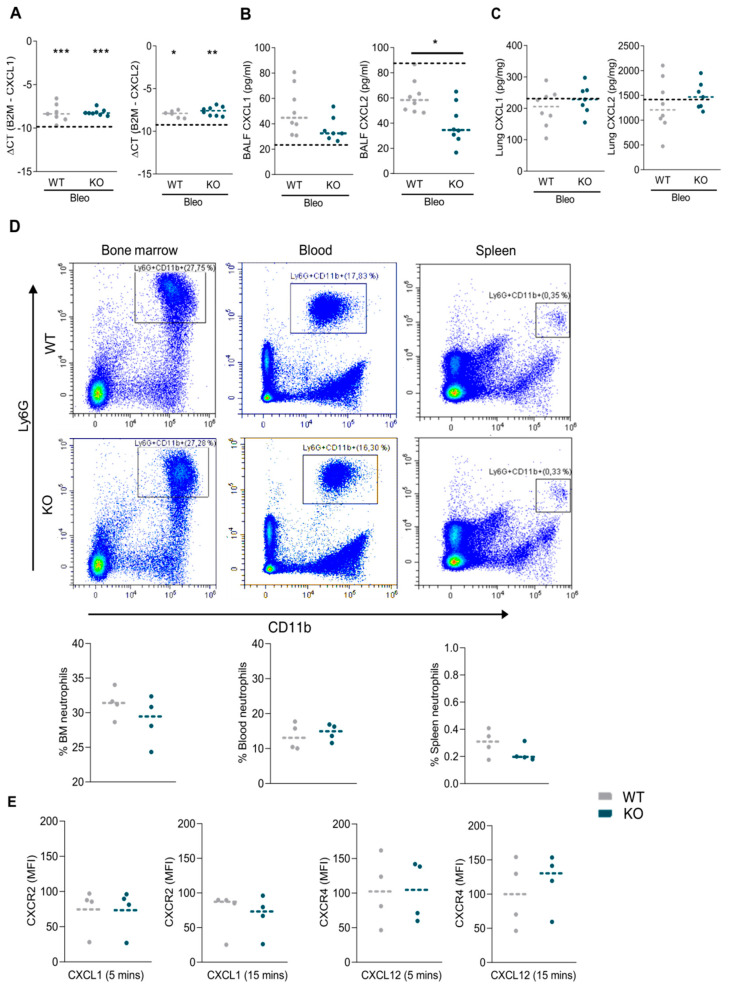
Characterization of RGS5-deficient neutrophils. Neutrophil recruitment-specific cytokine measurements in WT and RGS5-/- mice show (**A**) mRNA expression of CXCL1 and CXCL2 in lung tissue (*n* ≥ 7 mice/group). (**B**) CXCL1 and CXCL2 protein amount in BALF. ΔCT values, calculated by normalizing the expression of target genes to β2M expression, are shown. (*n* ≥ 7 mice/group). (**C**) CXCL1 and CXCL2 protein amount in lung tissue. Dotted lines represent mean of WT and RGS5-/- saline-treated pooled together, as no differences were observed. (*n* ≥ 7 mice/group). (**D**) Representative gated cell population from flow-cytometry for the assessment of Ly6G and cd11b positive neutrophils from bone marrow, blood and spleen with their respective quantification. (**E**) Assessment of change in CXCR2 and CXCR4 receptor expression on the neutrophils from WT and RGS5-/- mice after stimulation with CXCL1 and CXCL12 for 5 and 15 min measured via flow-cytometry. Four independent experiments were performed using 2–3 mice pooled for each genotype per experiment. Data are presented as scatter dot plots and analyzed using unpaired two-tailed *t*-test. The dashed lines in (**A**–**C**) show the means in control animals. * *p* < 0.05, ** *p* < 0.01 and *** *p* < 0.001 indicates the difference from saline treated animals.

The CXCL1 and CXCL2 mRNA levels in the lung tissue of bleomycin-treated mice were elevated in comparison to the saline-treated controls (Figure 5A). In BALF, CXCL2 was significantly reduced in RGS5-/- versus WT, and CXCL1 showed a similar tendency (Figure 5B). The CXCL1 and CXCL2 protein levels were not different in the lung tissue (Figure 5C). Neutrophil counts in the blood, spleen, and bone marrow under baseline conditions did not show significant differences between RGS5-/- and WT (Figure 5D). The presence of RGS5 in the neutrophils isolated from WT mice is shown in Supplementary Figure S5. The receptor levels on neutrophils (CXCR2 and CXCR4) under unstimulated conditions, or after stimulation with CXCL1 and CXCL12, were similar between neutrophils isolated from RGS5-/- and WT (Figure 5E). 

**Figure 6:**
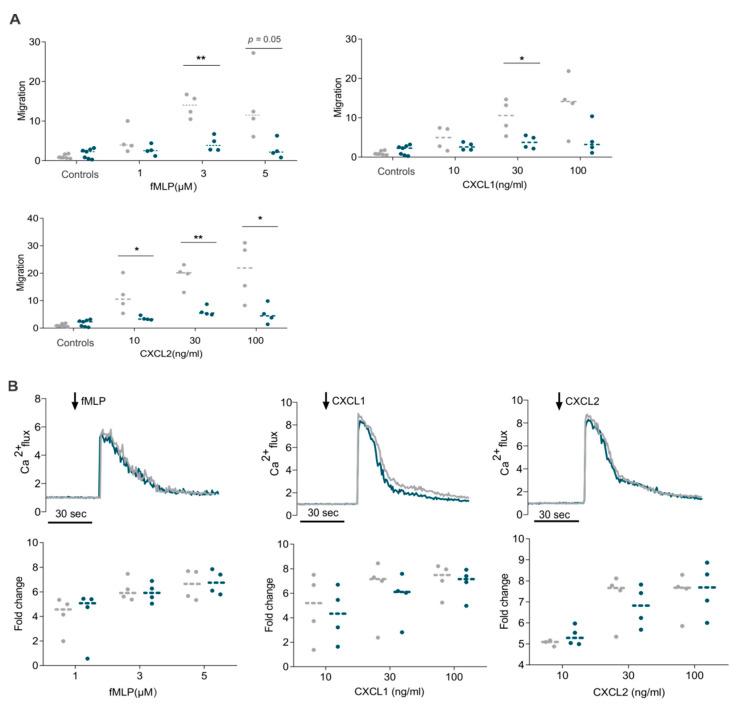
Impaired migration of neutrophils obtained from RGS5-/- animals. (**A**) Chemotaxis of neutrophils assessed by Transwell assays using fMLP, CXCL1, CXCL2. Minimum four independent experiments were performed using 2–3 mice pooled for each genotype per experiment. (**B**) Intracellular Ca^2+^ response of neutrophils upon stimulation. Peak intracellular Ca^2+^ response to chemoattractants fMLP, CXCL1 and CXCL2. The values were calculated as fold-change over baseline with respective histograms. Minimum four independent experiments were performed using 2–3 mice pooled for each genotype per experiment. Data are presented as scatter dot plots and analyzed using unpaired two-tailed *t*-test, * *p* < 0.05; ** *p* < 0.01.

Spontaneous migration of neutrophils obtained from WT and RGS5-/- animals did not differ. However, RGS5-/- neutrophils revealed a markedly impaired chemotactic motility compared to WT, independent of the kind of chemoattractant (Figure 6A). These findings demonstrate a function of RGS5 to modulate the migratory behavior of neutrophils. Classical GPCR signaling in neutrophils prominently involves mobilization of free cytoplasmic Ca^2+^. In our study, the Ca^2+^ levels were considerably elevated in neutrophils upon activation with fMLP, CXCL1 and CXCL2. The intracellular Ca^2+^ levels of RGS5-deficient neutrophils peaked in the same range and declined as in WT, indicating preserved Ca^2+^ signaling in RGS5-deficient neutrophils (Figure 6B). 

**Figure 7:**
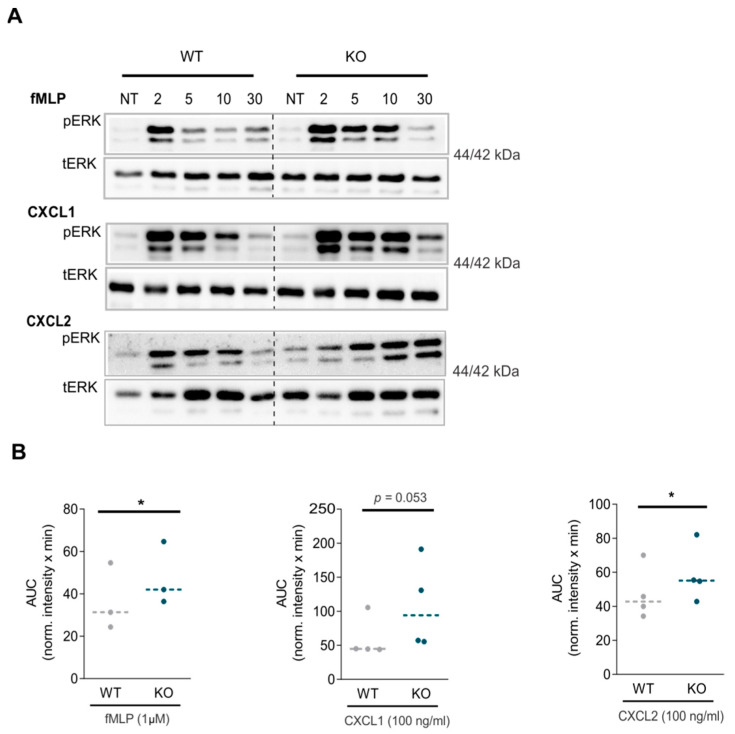
Effect of RGS5 loss on downstream signaling. (**A**) Representative blots of ERK phosphorylation and total protein expression in neutrophils isolated from WT and RGS5-/- mice. The neutrophils were stimulated with fMLP, CXCL1and CXCL2 for indicated time points. pERK values were internally normalized to the band intensity of total ERK at 2, 5, 10 and 30 min. NT = non-treated. 3–4 representative blots per chemoattractant. The 3–4 independent experiments were performed using 2–3 mice pooled for each genotype per experiment. (**B**) Summarized AUC values for all chemoattractants for both genotypes. Values represent normalized intensity x minutes. Data are presented as scatter dot plots and analyzed using paired *t*-test, * *p* < 0.05.

Further investigation of the downstream pathways revealed that activation of ERK seems to be more persistent in neutrophils obtained from RGS5-/- compared to WT. (Figure 7A). Upon fMLP and CXCL2 stimulation, more pERK could be detected in RGS5-/- neutrophils (Figure 7B and Figure S8). 

### 2.5. Absence of RGS5 Does Not Protect from Chronic Lung Fibrosis 

**Figure 8:**
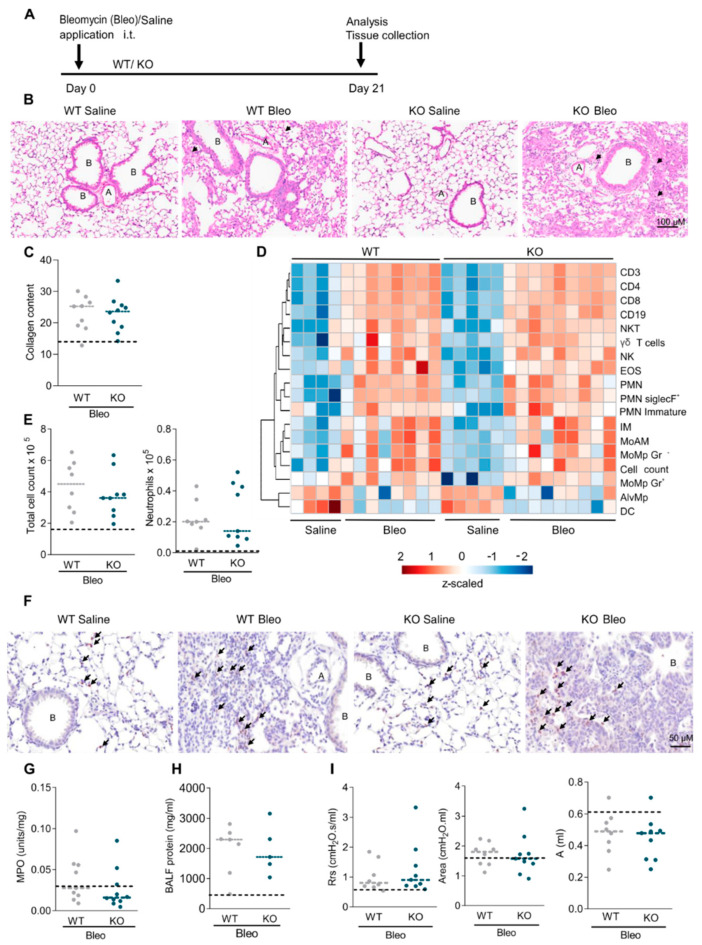
Absence of RGS5 does not prevent bleomycin-induced injury at day 21. (**A**) Schematic overview of the bleomycin treatment. (**B**) H&E staining, arrows indicating inflammation and distorted lung structure; scale bar: 100 µM. A = pulmonary arteries, B = bronchioles. (*n* ≥ 4 mice/group). (**C**) Lung collagen content determined via Sirius red staining quantification. (*n* ≥ 9 mice/group). (**D**) Heatmap presenting flow-cytometryanalyzed inflammatory cell populations in BALF at 21 days after saline and bleomycin-challenge (log_10_(x + 1) transformed, z-scaled per cell type) are shown with average distance sorted dendrograms from hierarchical clustering analysis. (*n* = 4–9 mice/group). (**E**) Total cell count and neutrophil numbers from BALF. (*n* ≥ 8 mice/group). (**F**) Anti-Ly6G IHC staining for neutrophils in lung tissue, arrows indicating neutrophils; scale bar: 50 µM. (*n* ≥ 3 mice/group). (**G**) MPO activity in lung tissue. (*n* ≥ 9 mice/group). (**H**) BALF protein measurements using BCA assay. (*n* ≥ 5 mice/group). (**I**) Lung function measurements showing the airway resistance (Rrs), area (enclosed under P-V loop), estimation of inspiratory capacity (**A**). Dotted lines represent means of pooled WT and RGS5-/- saline-treated animals. The dashed lines in (**C**,**E**,**G**–**I**) show the means in control animals. Data are presented as scatter dot plots (*n* ≥ 8 mice/group) and analyzed using unpaired two-tailed *t*-test.

We finally investigated the in vivo impact of RGS5 on chronic lung fibrosis on day21 after bleomycin treatment (Figure 8A). H&E staining showed distortion of the lung structure of both WT and RGS-/- as compared to saline-treated mice (Figure 8B). The same was true for the histologic analysis of fibrosis, showing extensively remodeled lung tissue with increased staining for Picrosirius red (Figure 8C and Figure S6A). Heatmaps of the myeloid and lymphoid immune cells in BALF and lung tissue of both genotypes showed that the total cell count and neutrophil recruitment were increased in BALF after bleomycin treatment (Figure 8D and Figure S6B). However, there were no differences between WT and RGS5-/- (Figure 8E). Ly6G staining and MPO activity assay on the lung tissue showed only a slight increase in neutrophil numbers and persistent alveolar leakage in both genotypes (Figure 8F–H). Lung function was similarly worsened upon bleomycin challenge in both WT and RGS5-/- (Figure 8I and Figure S6C). 

## 3. Discussion

Targeting neutrophil recruitment in the lung is an important element for developing anti-inflammatory therapies, and modulation of the innate immune response and the regulators of G-protein signaling (RGS) are emerging targets for novel therapeutics. The present study shows the intricate connection between RGS5 and neutrophil hyperinflammation, with unexpected outcomes. The absence of RGS5 resulted in less lung function deterioration and a blunted inflammatory response during the acute phase of bleomycin-induced and LPS-induced acute lung injury. This was mainly due to attenuated neutrophilic recruitment into the lung. Furthermore, in neutrophils obtained from RGS5-/- animals, delayed dephosphorylation of ERK was detected. 

The best understood function of RGS proteins is to reduce signaling output from GPCR activation. Although a significant number of studies have investigated the R4 subfamily, our knowledge about its function in the lung has remained limited. To the best of our knowledge, the current study is the first to show the differential regulation of the R4 subfamily in human fibrosing lung disease. We detected RGS5 in inflammatory cells and in lung structural cells both in the acute inflammatory and the fibrosing phases of the disease, indicating that our finding might be important for inflammatory lung diseases. Inflammation, as an essential pathological mechanism of both stages of the disease, has been discussed for a long time [11,12,13] and recent studies have demonstrated elevated chemokines and inflammatory cells, including neutrophils in the parenchyma and BALF of the patients [14,15,16,17,18]. However, broad anti-inflammatory approaches have not been successful in treating the progression or in showing any benefit on overall survival in ILD [19,20,21]. Therefore, selective targeting of RGS5 to prevent acute responses to inflammatory stimuli might represent a suitable approach in this context. 

In two independent animal models, bleomycin- and LPS-induced acute lung inflammation, we show that RGS5 loss attenuates lung function disorders in the acute inflammatory phase which can be explained by blunted neutrophil recruitment. We have explored the underlying molecular mechanism utilizing both in vitro and in vivo experimental models, thus extending the current knowledge. According to previous studies from the literature, overexpression of RGS5 in airway smooth muscle cells reduced the airway smooth muscle contraction in vitro and RGS5 deficiency resulted in spontaneous airway hyperresponsiveness (AHR) in vivo or in an increased airway neutrophilic inflammation upon viral infection [6,22,23]. In contrast to RGS5 knockout, RGS2-/- mice displayed increased acute inflammation upon house dust mite and LPS inoculation and an augmented development of pulmonary fibrosis [4,7,24]. Increase of RGS4 in human bronchial smooth muscle cells caused severe airway obstruction and RGS4-/- mice exhibited diminished allergen-induced AHR through the increased secretion of the bronchodilator PGE2 [25,26]. This suggests that the signaling pathways and biological roles of the different RGS proteins may be quite specific.

We detected significant attenuation in neutrophil numbers in the lung and the BALF and reduced MPO activity in the lung of RGS5-/- animals, after both bleomycin and LPS-induced lung injury. RGS5-/- neutrophils demonstrated restricted migration toward chemoattractants suggesting that RGS5 is required for directed migration of the neutrophils. As neutrophils are key mediators of host defense responses at the earliest stages of injury, migrating rapidly through tissues to reach sites of infection and tissue damage, investigations of neutrophil responses in inflammatory lung diseases have attracted considerable interest over the last decade. In acute lung injury, increased neutrophil numbers and the chemotactic factors that drive neutrophil recruitment are associated with increased disease severity and higher mortality rates [17,18]. Neutrophils are also increased in BALF from patients with idiopathic lung fibrosis, and this has been associated with early mortality [27]. Furthermore, studies investigating the progression of lung fibrosis showed that neutrophil elastase activates TGF-ß and recruits inflammatory cells to the lung, thereby promoting pulmonary fibrosis [28,29,30]. These findings suggest that the neutrophils are central to driving the inflammatory state. Thus, fine-tuning neutrophil recruitment to inflammatory sites could represent an effective tool for the development of anti-inflammatory therapies.

RGS regulates the Ca^2+^ homeostasis of the neutrophils by controlling the activation of GPCR. Thus we investigated this critical step in RGS5-/- animals. Our study shows that changes in cytosolic Ca^2+^ concentration upon receptor activation remained intact in RGS5-/- neutrophils. Considering the central role of intracellular Ca^2+^ signaling for various cellular processes, our findings of targeting acute neutrophilic infiltration via RGS5 might provide a highly selective approach. 

Contrary to our findings, a previous study reported increased RGS5-/- neutrophil migration and increased Ca^2+^ flux upon receptor activation [23]. However, there are significant differences between the two studies, e.g., different RGS5-/- lines and assays. Chan et al. used an RGS5-/- mouse model which was generated by replacing parts of exon 1 and intron 1 of the RGS5 gene with a dual cassette containing lacZ. These mice have significantly fewer leucocytes/lymphocytes which can already be the result of impaired lymphocyte trafficking [23,31]. The vascular cells from these mice exhibit exaggerated phosphorylation of ERK at rest and upon stimulation [31]. In addition, Chan et al. incubated the isolated neutrophils overnight with granulocyte/macrophage colony-stimulating factor (GM-CSF) before performing the assays. We used an RGS5-/- mouse model which was generated via specific deletion of the entire RGS domain of RGS5 [32] and the isolated neutrophils were used for in vitro assays after isolation without overnight stimulation. This might explain the different outcomes of the studies. 

### ERK

As ERK has been implicated in the regulation of neutrophil migration, we explored the ERK phosphorylation level and found more pERK in neutrophils from RGS5-/- mice upon different chemoattractants compared to WT, which might be a hint for delayed dephosphorylation (inactivation) of ERK. ERK involvement in neutrophil chemotaxis has been controversial, but more recent studies by Liu and co-workers support our hypothesis that ERK activation inhibits neutrophil migration [33]. Using the HL-60 cell line, this study identified p38 and ERK as two opposite regulators of neutrophil migration. fMLP treatment of the cells above 500 nM led to a significant decrease in cell migration, and increasing concentrations of fMLP resulted in a plateau of ERK activation. Treatment with an ERK inhibitor or ERK RNAi restored cell migration towards the chemoattractant [33]. At this point, it is challenging to interpret the correlation between activated ERK and impaired migration of RGS5-/- neutrophils. Further investigations using specific inhibitors of ERK and exploring the downstream pathways will be required to understand the role of ERK activation in this scenario.

Interestingly, in contrast to the acute models, the chronic animal study revealed no difference in the development of lung fibrosis between WT and RGS5-/- mice, although we still detected an increased expression of RGS5 in the chronic fibrotic lungs from mice and in human ILD lungs. It is possible, that RGS5 has different roles in different stages of the disease. In the acute phase, it seems to be responsible for inflammatory cell migration. In the chronic phase, without acute inflammation, RGS5 could be also detected in lung structural cells in the human samples. 

Our study has several limitations. We utilized human lung tissue samples from patients with ILD and healthy donors (controls) as a means to investigate RGS5 expression. However, the ILD samples were obtained from end-stage lung diseases. Thus, further studies are needed to define the role of RGS5 in the development of interstitial lung disease, or for spontaneous or infection-triggered flare-ups. The other drawback is that we employed murine models of acute lung injury and fibrosis. Although non-physiological stimuli induce these models, they still provide the backbone for most preclinical studies of fibrosing lung diseases. Accordingly, further investigations on the relevance of RGS5 in acute pulmonary inflammation and acute lung injury in the clinical setting are needed. Despite all these limitations we believe there is sufficient evidence to say that RGS5 is a valuable therapeutic target for fibrosing lung disease.

In conclusion, our studies have shown that lack of RGS5 is strongly associated with preserved lung function and reduced neutrophilic hyperinflammation in two independent animal models of acute lung injury. The significant effects of RGS5 on the cellular and functional level might provide a rationale for a novel therapeutic approach to treat acute exacerbations in fibrosing lung diseases, without compromising anti-infection defense mechanisms. 

## 4. Materials and Methods

### 4.1. Human Lung Samples

We used lung samples from ILD patients undergoing lung transplantation (Department of Surgery, Division of Thoracic Surgery, Medical University of Vienna, Austria) or from patients with ARDS (Department of Internal Medicine, Justus-Liebig-University Giessen, Germany) from 2017 to 2020. The study protocols for tissue donation were approved by the institutional ethics committees of the Medical University of Vienna (IRB approval No 976/2010) and the Faculty of Medicine, Justus-Liebig-University, Giessen, Germany (IRB appoval No 29/01) following national law and with Good Clinical Practice/International Conference on Harmonization guidelines. As controls we used donor lungs that had to be down-sized. Thus, samples from healthy lungs are referred to as controls in our study. Written consent was obtained from all study participants or their next-of-kin. For ARDS, all patients met clinical American–European Consensus Conference criteria and died in the early phase, with a mean duration of mechanical ventilation of 92 h. Experienced pneumologists and pathologists extensively reviewed and confirmed the diagnosis of all lungs. Healthy controls and patient characteristics are given in Supplementary Tables S1 and S2.

### 4.2. Animal Models and Treatments

RGS5 knockout (RGS5-/-) mice were generated previously at the Division of Matrix Biology, in the Department of Medical Biochemistry and Biophysics, Karolinska Institute, Stockholm, Sweden [32]. All animal studies were approved by the local authorities according to national regulations (Austrian Ministry of Education, Science and Culture (BMWF-66.010/0049-WF/V/3b/2017, BMBWF-66.010/0142-V/3b/2019)). The treatments were performed as described previously [34,35]. The details are given in Supplementary Materials and Methods.

### 4.3. Lung Function Testing

Lung function measurements of the mice were performed via the Flexi Vent system (SCIREQ Inc, Montreal, QC, Canada) as described previously [35]. The details are given in Supplementary Materials and Methods.

### 4.4. Sample Collection and Preparations

Broncho-alveolar lavage fluid (BALF) was collected separately, once with 1 mL and once with 4 mL BAL buffer (PBS containing protease inhibitor cocktail (Thermofischer, Rockford, USA) and 1 mM EDTA). After BALF spin-down (300 g at 4 °C for 8 min) and total cell count assessment, samples were used for flow cytometry analysis. Supernatant from 1 mL BALF was stored for further total protein and chemokine measurement. Lower right lobes of lung collected from animal experiments were homogenized to prepare single cell suspensions as previously described [36] and were used for immune cell analysis via flow cytometry. 

### 4.5. Flow Cytometry

BALF and lung tissue single cells were stained with myeloid and lymphoid antibody panels shown in Supplementary Table S3. Cells were subdivided in the following cell types/groups—myeloid: PMN (neutrophils) (CD11b^+^, Gr-1^+^, siglecF^+/−^), PMN immature (immature neutrophils) (CD11b^+^, Gr-1^+^), PMN siglec F^+^ (siglec F^+^ neutrophils) (CD11b^+^, Gr-1^+^, siglecF^+^), alveolar macrophages (AlvMp) (CD11b^+/−^, CD11c^+^, siglecF^+^, CD64^+^), intersitital macrophages (IM) (CD11b^+/−^, CD11c^−^, siglecF^−^, MHC-II^+^, CD64^+^), monocyte macrophages Gr^+^ (MoMp) (CD11b^+^, CD64^+^, Gr^+^), monocyte macrophages Gr^+^ (MoMp) (CD11b^+^, Gr^−^), monocyte-derived macrophages (MoAM) (CD11b^+/−^, CD11c^+^, Siglec F^Low^, CD64^+^), eosinophils (EOS) (CD11b^+^, CD11c^−^, Siglec F^+^) and dendritic cells (DC) (CD11b^+^, Gr-1^−^, MHC-II^High^). Lymphyoid: non-class T cells (CD3^+^), T helper cells (CD3^+^, CD4^+^), cytotoxic T cells (CD3^+^, CD8^+^), γδ T cells (CD3^+^, CD4^+^, γδTCR^+^), B cells (CD19^+^), natural killer T cells (NKT) (CD3^+^, NK1.1^+^) and natural killer (NK) (CD3^−^, NK1.1^+^). LSRII flow cytometer (BD, Biosciences, Vienna, Austria) and cytoFLEX-SII (Beckman Coulter, Vienna, Austria) were used to count the stained cells and FACSDiva software (BD, Biosciences, Vienna, Austria) and Flowjo v10 (LLC, Ashland, OR, USA) [37] for analysis. The distribution of the myeloid and lymphoid cells from the wild type animals of this study has been incorporated into a summary of our scientific work [38].

### 4.6. Myeloperoxidase (MPO) Assay

The MPO activity measurements were performed as described previously [39]. The details are given in Supplementary Materials and Methods.

### 4.7. BCA Assay

Total protein concentrations of BALF and lung tissue homogenates were determined with the Bicinchoninic acid assay (BCA) and performed as suggested by the manufacturer (Thermofischer, Rockford, IL, USA).

### 4.8. Haemodynamic Measurements

The haemodynamic measurements were performed as described previously [40]. The details are given in Supplementary Materials and Methods.

### 4.9. Isolation of Neutrophils

Neutrophils were isolated from bone marrow of WT and RGS5-/- mice using the anti-Ly6G positive selection kit (Miltenyi Biotec, Bergisch Gladbach, Germany). After each isolation, neutrophils were rested for 30 min at room temperature (RT) before employing any neutrophil-specific assay. The details are given in Supplementary Materials and Methods.

### 4.10. Neutrophil Migration Assay

The neutrophil chemotaxis assay was performed using trans-well plates (Corning, 5 µm pore size). The trans-wells were pre-incubated with 1% BSA in PBS for 30 min at 37 °C and washed with assay buffer. After neutrophil purification (anti-Ly6G beads positive selection), 20,000 cells in 100 µL were seeded in the upper compartment and allowed to migrate for 60 min at 37 °C toward different concentration of chemoattractants (110 µL of assay buffer with fMLP (F3506, Sigma Aldrich, Saint Louis, MO, USA), CXCL1 (250-11, Pepprotech, Cranbury, NJ, USA) and CXCL2 (250-15, Pepprotech, Cranbury, NJ, USA), which were placed in the lower compartment of the trans-well chamber. Non treated cells were used as control. Neutrophils were collected from the lower compartment and resuspended in 150 µL of fixative solution for flow cytometry cell counting. 

### 4.11. Calcium (Ca^2+^) Flux Measurement

The isolated neutrophils were re-suspended in 2 μM fluo-3-acetoxymethyl ester (Ca^2+^-sensitive dye) (Thermofischer, Waltham, MA, USA) and 0.02% Pluronic F-127 (Thermofischer, Waltham, MA, USA) for 1 h at RT in the dark. Baseline response was recorded for 60 s before addition of any agonist. As the agonist to elicit the Ca^2+^ response in the neutrophils, different concentrations of the chemoattractants fMLP, CXCL1 and CXCL2 were applied. Intracellular Ca^2+^ level differences were quantified in fluo-3 positive cells in FL1- channel via a FACSCalibur flow cytometer (BD, Franklin Lakes, NJ, USA). The Ca^2+^ response for each agonist was corrected to its own baseline.

### 4.12. Neutrophil Count and Receptor Analysis

Blood, spleen and bone marrow of WT and RGS5-/- was collected and stained with Ly6G and CD11b. All single staining controls were included for comparison. The details of antibodies in Supplementary Table S4 and procedures are depicted in Supplementary Material and Methods.

### 4.13. Immunohistochemical Stainings

Lung tissue sections from human and mouse were stained with anti-RGS5 and Ly6G antibodies. The details of antibodies are given in Supplementary Table S4 and procedures are depicted in Supplementary Material and Methods.

### 4.14. RNA Isolation and RT-qPCR

Isolation of total RNA from lung tissues was performed according to manufacturer’s instructions using peqGOLD Total RNA Kit (Peqlab, Erlangen, Germany). The details of primers in Supplementary Table S5 and procedures are given in Supplementary Material and Methods.

### 4.15. Protein Isolation and Western Blotting

For protein isolation, lung tissues and neutrophils were lysed with CHAPS (Tocris, Bristol, UK) or RIPA (Sigma, Saint Louis, MO, USA) buffer containing EDTA and protease inhibitor cocktail

**Figure 9:**
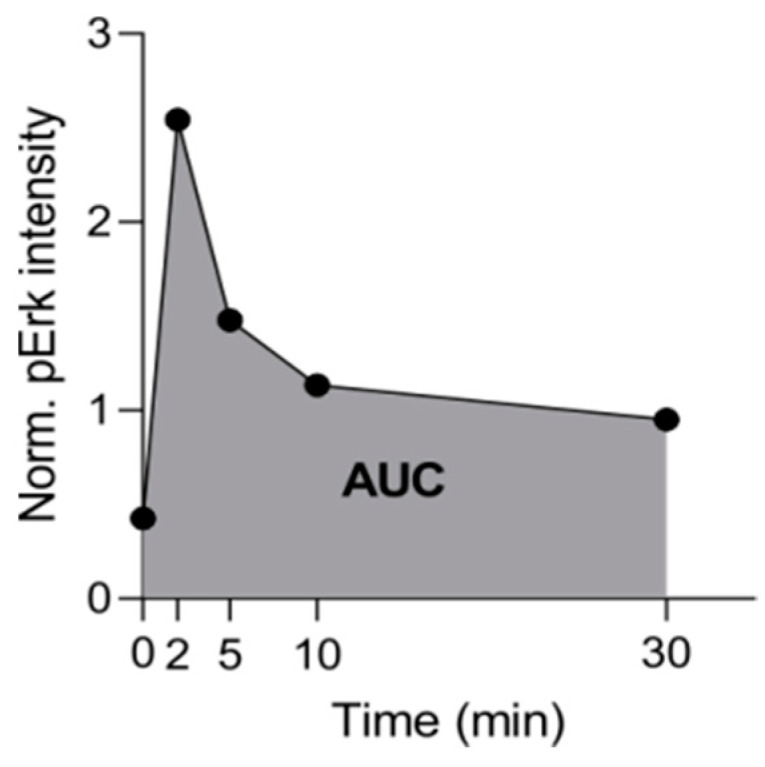
Representative scheme addressing quantification of total pERK throughout the detection period.

Isolated neutrophils (300,000 cells/condition) were stimulated with fMLP (1 µm), CXCL1 (100 ng/mL), and CXCL2 (100 ng/mL) for the indicated times (2, 5, 10 and 30 min). Cell pellets were washed with PBS and resuspended after centrifugation in radioimmunoprecipitation lysis buffer containing protease and phosphatase inhibitors, for protein extraction. Further details on western blotting procedure are provided in Supplementary Material and Methods. The details of antibodies are given in Supplementary Table S4. The calculation for pERK values was performed via internally normalized to the band intensity of tERK at 2, 5, 10 and 30 min. For quantification of pERK for the same time period (relative band intensities versus time), the area under the curve (AUC) was calculated [41,42]. This representation of the data allows quantification of total pERK throughout the detection period. The scheme is depicted below (Figure 9).

### 4.16. Statistical Analysis

Statistical analysis was performed in GraphPad Prism 8 (GraphPad Software, La Jolla, CA, USA) with details for each graph noted in the figure legend. Significance was defined by a *p* value less than 0.05. Data are presented as scatter dot plots with median. Dendrograms and heatmaps were created using R (version 3.6, R Core Team, Vienna, Austria) [38,43]. The dendrograms were clustered by Lance–Williams dissimilarity update with complete linkage (R function dist and hclust) and sorted (R function dendsort) at every merging point according to the average distance of subtrees and plotted at the corresponding heat maps (R function heatmap).

## Data Availability

Not applicable.

## Supplementary Materials

The following are available online at https://www.mdpi.com/article/10.3390/ijms22179342/s1.
