# Supplementary material for: RGS5 Determines Neutrophil Migration in the Acute Inflammatory Phase of Bleomycin-Induced Lung Injury
Source: Int J Mol Sci. 2021 Aug 28;22(17):9342. doi: 10.3390/ijms22179342 (PMC8430858; doi:10.3390/ijms22179342)
Supplement: Supplementary file 1 [file ijms-22-09342-s001.zip › Supplementary Figure methods_PR.pdf]

Supplementary materials and methods

Animal models and treatments

The mice from age of 12-16 weeks were used in our acute and chronic treatment conditions. Experimental animals were kept under controlled temperature and 12 hours dark-light cycle with water and chow diet. All the experimental procedures described further were performed under anesthesia using a continuous flow of isoflurane (2%). At the end of treatment, after haemodynamic measurements and lung function testing, mice were sacrificed for further sample collection, immune evaluations, including FACS analysis of the Broncho alveolar lavage fluid (BALF), and lung tissue. Molecular studies, such as gene expression analysis and western blotting were performed on isolated lung tissue. Intra-tracheal (i.t.) administration of bleomycin sulphate (Sigma Aldrich, Vienna, Austria) was performed by using a microsprayer® Aerosoliser (Penn-Century. Inc. Wyndmoor, PA, USA) which takes ~5 seconds and results in a uniform distribution of aerosol throughout the lung.

Chronic treatment: wildtype (WT) littermate controls and RGS5-/- were treated with bleomycin sulphate (0.7 units/kg)/saline (i.t.) at day 0. The mice were kept under observation and were weighed every second day for the following 21 days until the end of the experiment.

Acute treatments: WT and RGS5-/- mice were challenged with bleomycin sulphate (1 unit/kg)/saline (i.t.) at day 0 and analysed at day 3. In separate animal experiments, mice were given intra-nasally (i.n.) 1 mg/kg of Lipopolysaccharide (LPS) (0111 Serra, Sigma Aldrich, Vienna, Austria) in saline or saline only. After 24 hours of treatment further physiological measurements were performed.

Haemodynamic measurements

WT and RGS5-/- mice underwent cardiac catheterization under constant anaesthesia using isoflurane (2%) for the ventricular pressure measurements. The mice were monitored continuously to obtain body temperature, heart beat and ventricular pressure using SPR-671 1.4 F catheters (Millar Instruments Inc., Houston, USA) with a sampling rate of 1 kHz, coupled to a Millar PCU-2000 pressure control unit and PowerLab 8/30 acquisition system (AD Instruments, Spechbach, Germany). Following an incision in the neck, the catheter was inserted into the right jugular vein and directed to the right ventricle to measure right ventricular systolic pressure (RVSP). Collected data were analysed through powerlab pro software. At the end of measurements, the mice were anesthetized intra-peritoneally (i.p.) with a mixture of Ketanest (150 mg/kg ketamine, Parke-Davis, Germany) and Rompun (20 mg/kg xylazine, Bayer, Germany) (ratio of 1:1.) before being transferred for lung function measurements.

Lung function testing

In order to connect mice trachea to the animal ventilator tube, a tracheostomy was performed. Lung function parameters were obtained from pressure-volume signals generated in response to perturbations. Single compartment model, constant phase model and pressure volume curve were used to calculate multiple functional parameters such as compliance (Crs), resistance (Rrs), elastance (Ers), tissue elastance (H), tissue damping (G), estimation of inspiratory capacity (A) and area. After the measurements, sample collection was performed.

Myeloperoxidase (MPO) assay

The snap frozen lung tissue of mice were weighed and homogenized in phosphate buffer (pH 6.0) with 0.5% hexadecytrimethyl ammonium bromide (Sigma Aldrich, Saint Louis, MO, USA) using ceramic beads and MagNA Lyser Rotor (Roche, Vienna, Austria). 10 µl of supernatant was added into the 96 well plates in triplicates. 200 µl of O-dianisidine buffer—50 mM potassium phosphate buffer (pH 6.0) containing 0.167 mg/ml of O dianisidine hydrochloride (Sigma Aldrich, Saint Louis, MO,USA) and 0.5 µl of 1% H_2O_2/ml— were added in each samples and measured at 460 nm using micro-plate reader (BMG, Vienna, Austria).

Isolation of neutrophils

After euthanizing the mice, hind limb bones were dissected and bone marrow was collected by flushing each bone with 1 ml of RPMI media. Collected bone marrow was spinned down (1100 rpm at room temperature (RT) for 7 mins). The obtained cell pellet was further incubated with 5 ml erythrolysis buffer for 5 mins on a rotor. The pellet was washed with 15 ml of RPMI media, spun down and supernatant was discarded to stop the erythrolysis reaction and to obtain a erythrocyte free cell pellet. Next steps for neutrophils isolation were performed according to manufacturer's instructions using the anti-Ly6G positive selection kit (Miltenyi Biotec, Bergisch Gladbach, Germany) and MACs buffer. In brief, Ly6G beads were mixed and incubated with the cell pellet at 4 °C for 10 mins and further washed and spun down (300 g at RT for 7 mins). The pellet was resuspended and passed through the LS column attached to the magnet stand (Miltenyi Biotec, Bergisch Gladbach, Germany). The purified neutrophils were collected and counted on Neubauer chamber using

trypan blue dye. After each isolation, neutrophils were rested for 30 mins at RT before employing any neutrophil-specific assay.

CXCL1 and CXCL2 ELISA

CXCl1 and CXCl2 were measured following the manufacturer's ELISA kit (DY453-05 and DY452-05; R&D systems, Minneapolis, MN, USA USA). For CXCl1 BALF samples were diluted to 1:2 and for CXCl2 undiluted samples were used. The standards and samples were corrected for blank and OD values obtained at 570 nm using micro-plate reader. All the unknown chemokine concentrations (pg/ml) were calculated using equations derived from standard curves.

Neutrophil count and Receptor analysis:

The details of antibodies are given in Supplementary table S4. Blood, spleen and bone marrow of WT and RGS5-/- mice were collected and stained with Ly6G and CD11b. All single staining controls were included for comparison. Bone marrow isolation was perfomed as described the in neutrophil isolation section. Blood: 50 µl of whole blood was taken for each condition, followed by 20 mins incubation in 20 µl of antibody master mix at RT. After adding 2 ml freshly prepared FACS lysis solution for 10 mins, samples were centrifuged (300 g at RT for 7 mins). The cell pellet was washed twice with 2 ml of MACS buffer and centrifuged again. Spleen: The spleen single cell suspension was prepared by cutting the tissue into small pieces and passing through a 100 µm cell strainer. The sample was collected and centrifuged (1100 rpm at 4 °C for 7 mins). The cell pellet was hemolysed as described previously in the neutrophil isolation section to further obtain a "erythrocyte free cell pellet". The cells were counted on a Neubauer chamber using trypan blue dye. Unstained and stained cell samples were mixed with 200 µl of MACS buffer and stored at 4 °C until

measurements. For the receptor level analysis, purified bone marrow derived neutrophils were either vehicle or CXCL1 (100 ng/ml) and CXCL12 (100 ng/ml; 250-20B, Pepprotech, Cranbury, NJ, USA) treated for 5 and 15 mins. Immediately fixative solution was added and transferred to ice. After centrifugation (8000 rpm at 4 °C for 2 mins) cells were stained for Ly6G, CD11b, CXCR2 and CXCR4.

Immunohistochemical stainings

Each lung tissue section (2.5 µm) was deparaffined overnight at 60 °C and rehydrated the next day (2 times 10mins xylol following 100%, 90%, 80%, 70%, 50% ethanol and finally aqua dest). Tissue slides were incubated with Proteinase K (Peqlab, Erlangen, Germany) solution for 10 mins at RT. Then, antigen retrieval solution of PH=9 (DAKO, Glostrup,Denmark) for anti-RGS5 and PH=6 (DAKO, Santa Clara, CA, USA) for anti- Ly6G was used and slides were incubated for 30 min at 95 °C and later cooled down to RT before continuing with antibody staining. For neutrophil anti-RGS5 staining, cytospin slides were fixed with formalin for 15 mins at RT and subsequently, incubated for 10 mins with 0.2% triton x-100 (Sigma, Saint Louis, MO, USA) at RT. To prevent unspecific binding, samples were incubated in MOM's kit (Vector Laboratories, Burlingame, CA, USA) blocking solution (only for RGS5 stainings on mouse samples) or 10%BSA in PBS for 1 hour at RT. Primary antibodies were incubated overnight at 4 °C; the following day washing steps were performed and secondary antibody labelling with peroxidase enzyme for 1 hour at RT. Stainings were developed using NOVA red and fast red (Vector Laboratories, Burlingame, CA and abcam, Cambridge, UK) . Nuclei staining was performed with haemalaun solution for 45 sec following dehydration steps (96%, 100% ethanol and xylol) and slide mounting using mount media (Thermofischer, MI,, USA). Negative

controls were involved in every protocol. The primary antibodies were omitted while processing the negative controls. The details of antibodies are given in table S4.

RNA Isolation and RT-qPCR

The concentration and purity of isolated total RNA was measured at 260 nm and absorbance ratio at 280/260 nm respectively using the Nanodrop 2000c spectrophotometer (ThermoScientific, Wilmington, Delaware,USA). Total RNA was reverse transcribed to cDNA using iScript cDNA Synthesis kit (Bio-Rad, Hercules, CA, USA). Real-time RT-PCR reaction measured the expression of Genes using QuantiFast SYBR Green PCR kit (Qiagen, Hilden, Germany) on a Light cycler 480 System (Roche Applied Science, Vienna, Austria). RT-PCR reaction mixtures contained transcribed cDNA, SYBR Green master mix, forward and reverse primers. Each sample was measured in duplicates. Melting curves were analysed after amplification to confirm the quality of the reaction. The targeted gene expression was calculated as ΔCT normalized to the level of beta-2 microglobulin (B2M) housekeeping gene, using the following formula ΔCT = CT (Housekeeping Gene) – CT (Gene of Interest). For ΔΔCT the following formula = ΔCT(test samples) – ΔCT(averaged controls). The sequences for primers are listed in supplementary table S5.

Protein isolation and western blotting

Lung tissues were homogenized using ceramic beads with MagNA Lyser Rotor following supernatant collection and centrifugation (10,000 rpm at 4 °C for 10 mins). Protein samples were separated by SDS-PAGE using 10-15% SDS gels (depending on protein-size of interest) and transferred to 0.45 μm nitrocellulose membrane

(GE Healthcare, Buckinghamshire, UK). Blocking was performed with 5% milk or 10% BSA for 1 hour at RT, followed by primary antibodies overnight incubation at 4 °C. The details of antibodies with dilutions used in this study are listed in Supplementary table S4. Next day membranes were incubated for 1 hour at RT with peroxidase-conjugated secondary antibodies. Membranes were incubated with ECL prime developing solution (GE Healthcare, Buckinghamshire, UK) and signal detection was done using a ChemiDoc Touch Imaging System (Bio-Rad, Hercules, CA, USA).
