# Supplementary material for: RGS5 Determines Neutrophil Migration in the Acute Inflammatory Phase of Bleomycin-Induced Lung Injury
Source: Int J Mol Sci. 2021 Aug 28;22(17):9342. doi: 10.3390/ijms22179342 (PMC8430858; doi:10.3390/ijms22179342)
Supplement: Supplementary file 1 [file ijms-22-09342-s001.zip › Supplementary Figure with Legends_PR.pdf]

Supplementary Figures

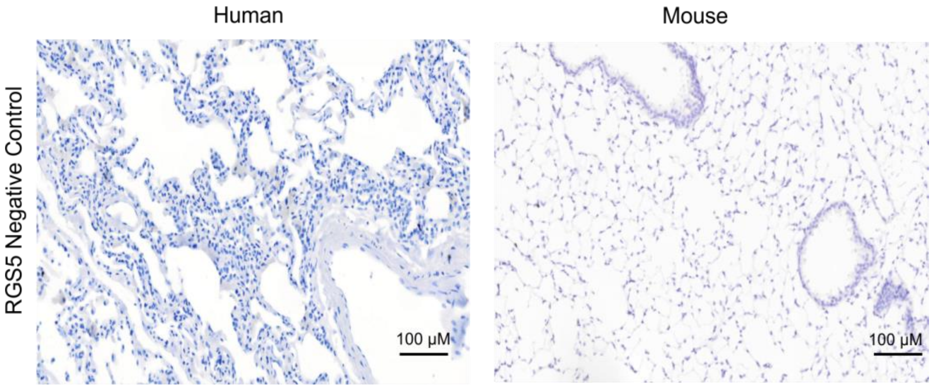

Supplementary Fig.S1 RGS5 IHC staining negative controls. Representative cross-sections are negative controls for RGS5 IHC staining in human and mouse lung tissue; Scale bar 100 μm.

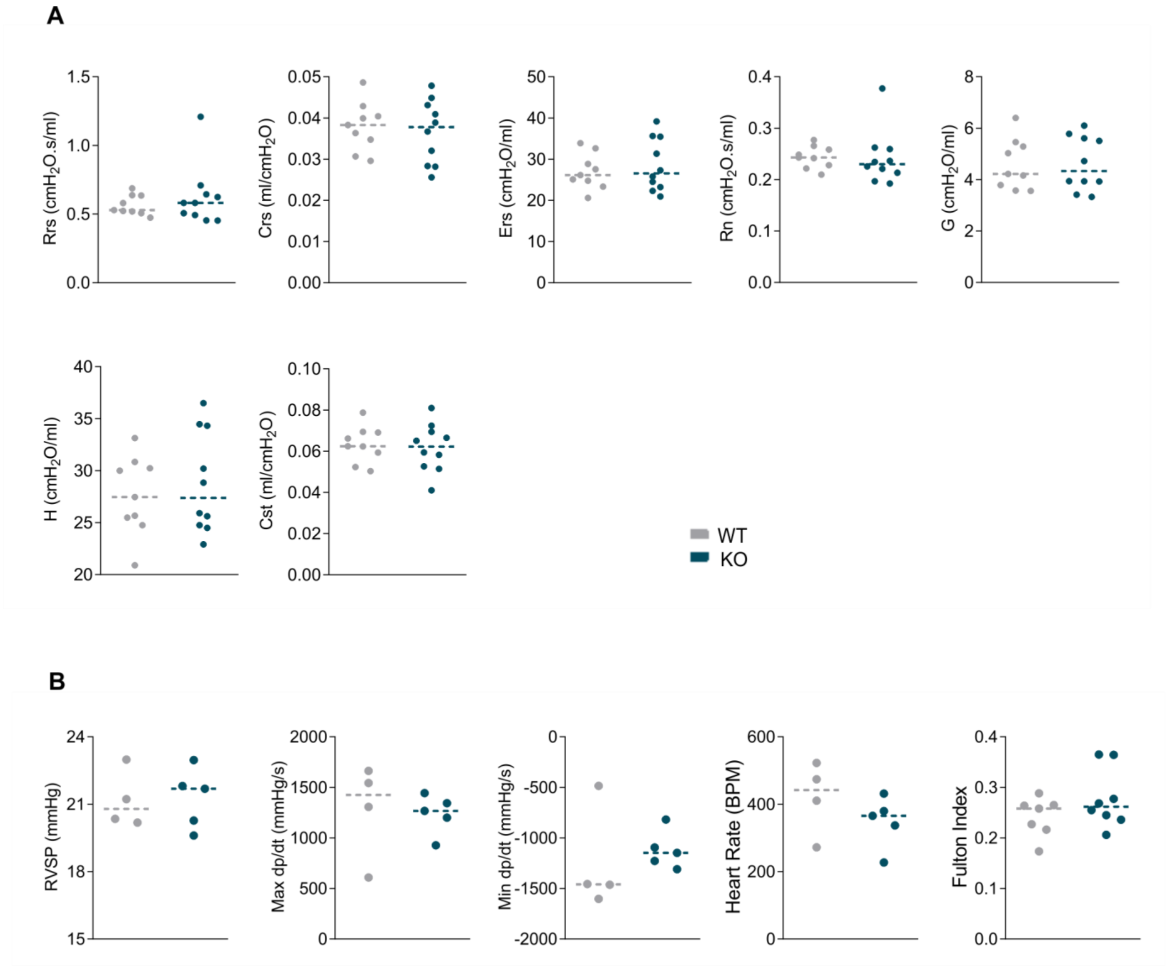

Supplementary Fig.S2 In vivo lung function and Hemodynamic characteristics of WT and RGS5-/- mice at baseline. (A) Lung function measurements of WT and RGS5-/- mice; Rrs: resistance; Crs: compliance; Ers: Elastance; Rn: central airway resistance; G: tissue damping; H: tissue elastance; Cst: quasi-static compliance of the respiratory system. (B) Hemodynamic measurements showing right ventricular systolic pressure (RVSP), max dp/dt, min dp/dt, heart rate (beats per minute) and Fulton index (RV/(LV+S)) measurements in WT and RGS5-/- mice. Data are presented as scatter dot plot (n=4-10) and analyzed using unpaired two-tailed t-test.

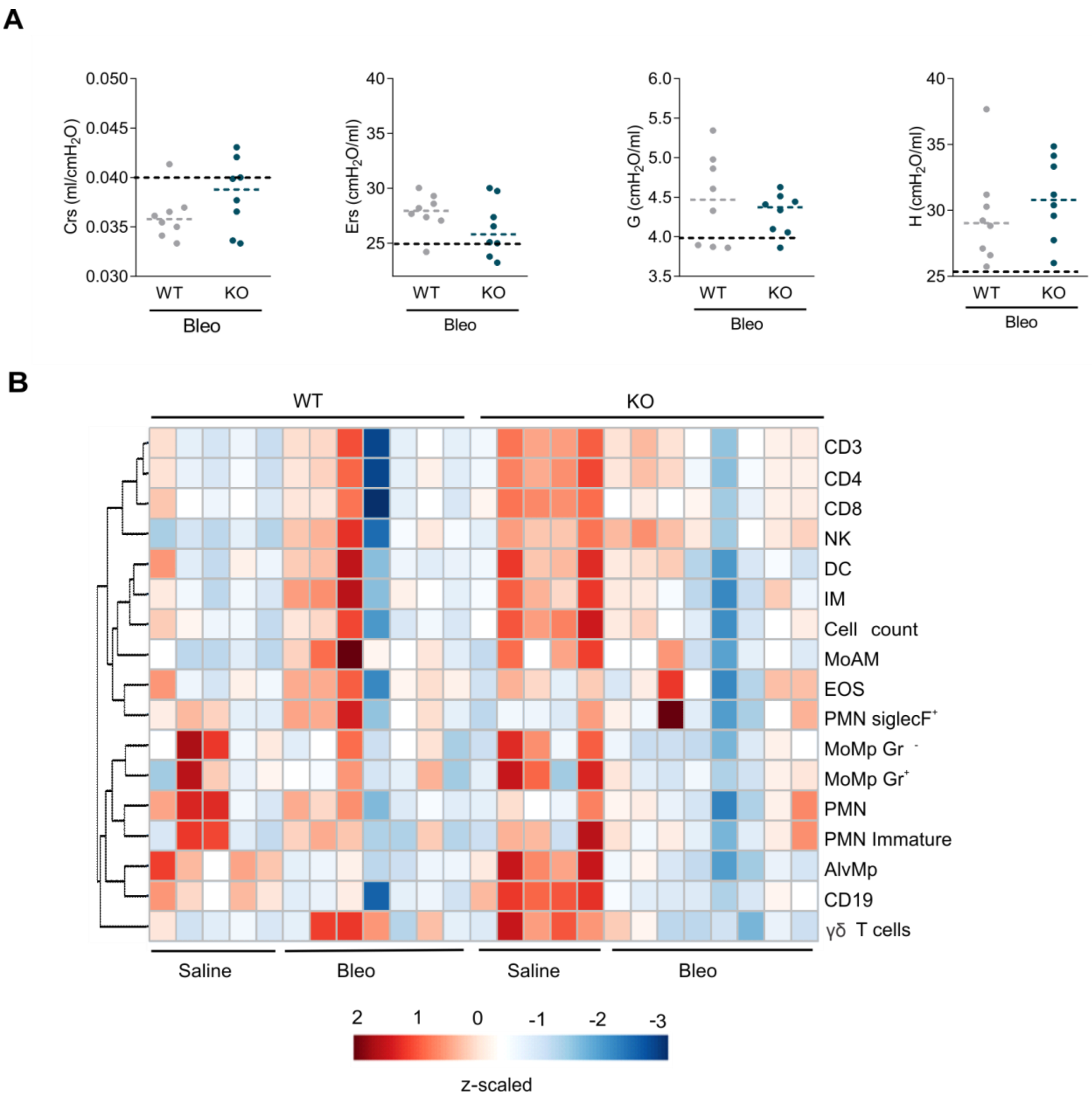

Supplementary Fig.S3 In vivo analysis of lung function and inflammatory cell after 3 days of bleomycin challenge. (A) Lung function measurements of WT and RGS5-/- mice showing Crs: compliance; Ers: Elastance; G: tissue damping; H: tissue elastance of the respiratory system. Dotted lines represent mean of WT and RGS5-/- saline treated pooled together, as no differences were observed. Data are presented as scatter dot plot (n=5-8) and analyzed using unpaired two-tailed t-test.(B) Heatmap presenting inflammatory cell populations obtained via flow-cytometry analysis in lung tissue of WT and RGS5-/- 3 days after saline and Bleomycin-challenge ($log_{10}(x+1)$

transformed, z-scaled per cell type) shown with average distance sorted dendrograms from hierarchical clustering analysis.

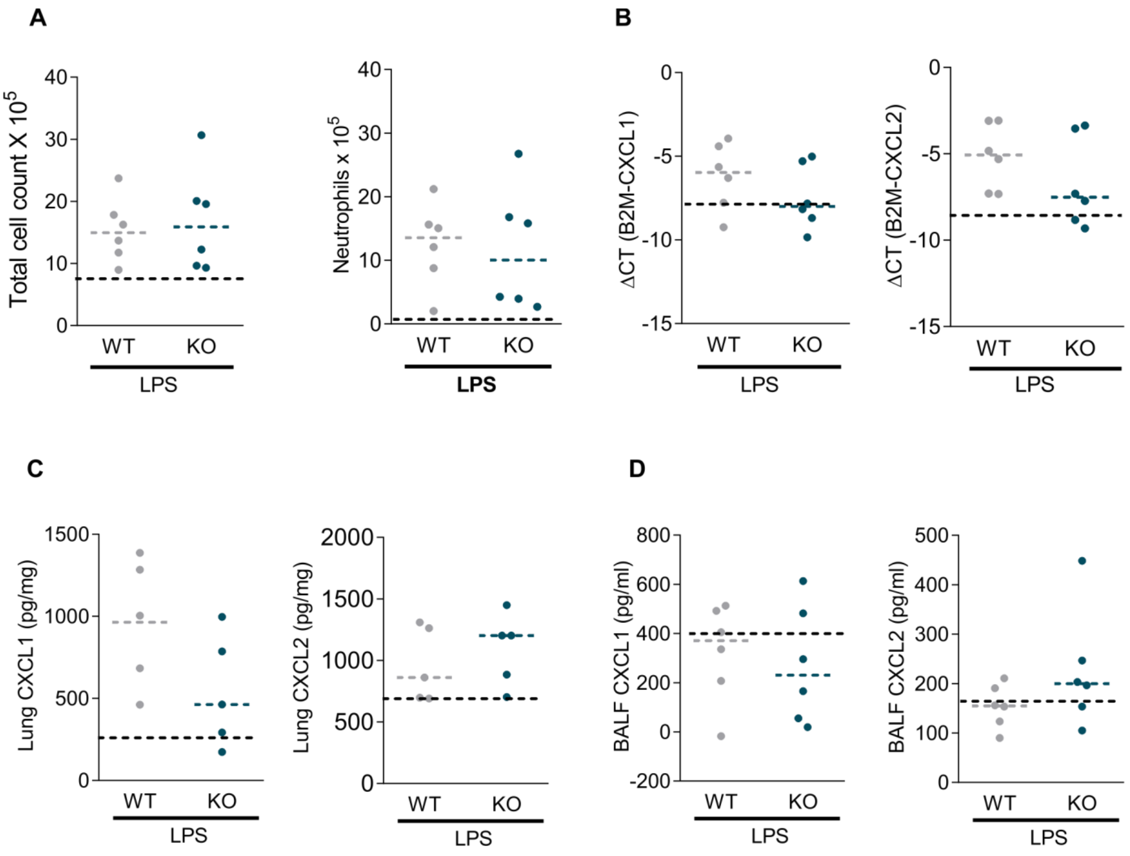

Supplementary Fig.S4 Neutrophil recruitment in BALF and measurement of neutrophil recruitment specific chemokines in WT and RGS5-/- mice challenged with LPS and saline. (A) Flow-cytometry analysis of BALF inflammatory cells to determine total cell count and neutrophils (B) mRNA expression of CXCL1 and CXCL2 in lung tissue. ΔCT values, calculated by normalizing the expression of target genes to β2M expression obtained via RT-qPCR. CXCL1 and CXCL2 protein levels are measured by ELISA in (C) lung (D) BALF. Dotted lines represent mean of WT and RGS5-/- saline-treated pooled together, as no differences were observed. Data are presented as scatter dot plot (n=4-6) and analyzed using unpaired two-tailed t-test.

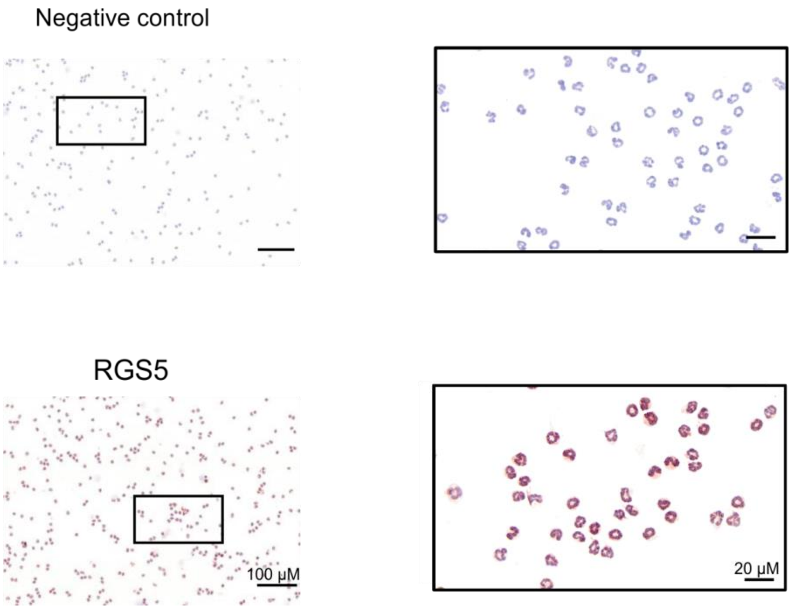

Supplementary Fig.S5 RGS5 expression in the neutrophils. RGS5 IHC staining (brown) on the neutrophils isolated from bone marrow of WT mice presented with their negative control. Scale bar 100 μm, 20 μm.

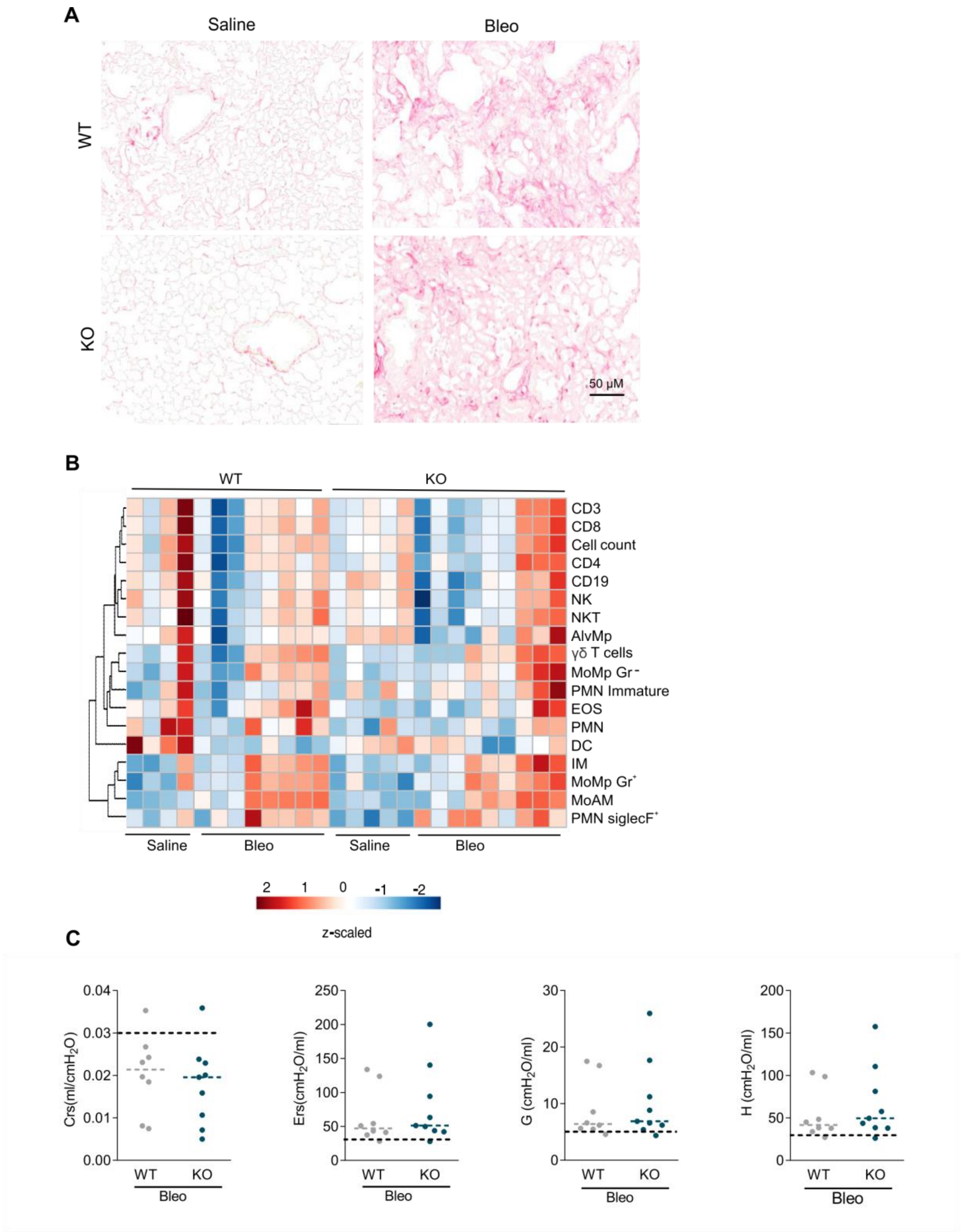

Supplementary Fig.S6 In vivo analysis of the lung after 21 days of bleomycin injury. (A) Lung collagen content determined via sirius red staining in WT and RGS5-/- lung tissue sections; Scale bar 50 μM (B) Heatmap presenting the inflammatory profile of lung tissue ($log_{10}(x+1)$ transformed, z-scaled per cell type) are shown with average distance sorted dendrograms from hierarchical clustering analysis at 21 days

obtained from flow-cytometry analysis (C) Lung function measurements of WT and RGS5-/- mice showing; Crs: compliance; Ers: Elastance; G: tissue damping; H: tissue elastance of the respiratory system. Dotted lines represent mean of WT and RGS5-/- saline treated pooled together, as no differences were observed. Data are presented as scatter dot plot (n=4-10) and analyzed using unpaired two-tailed t-test.

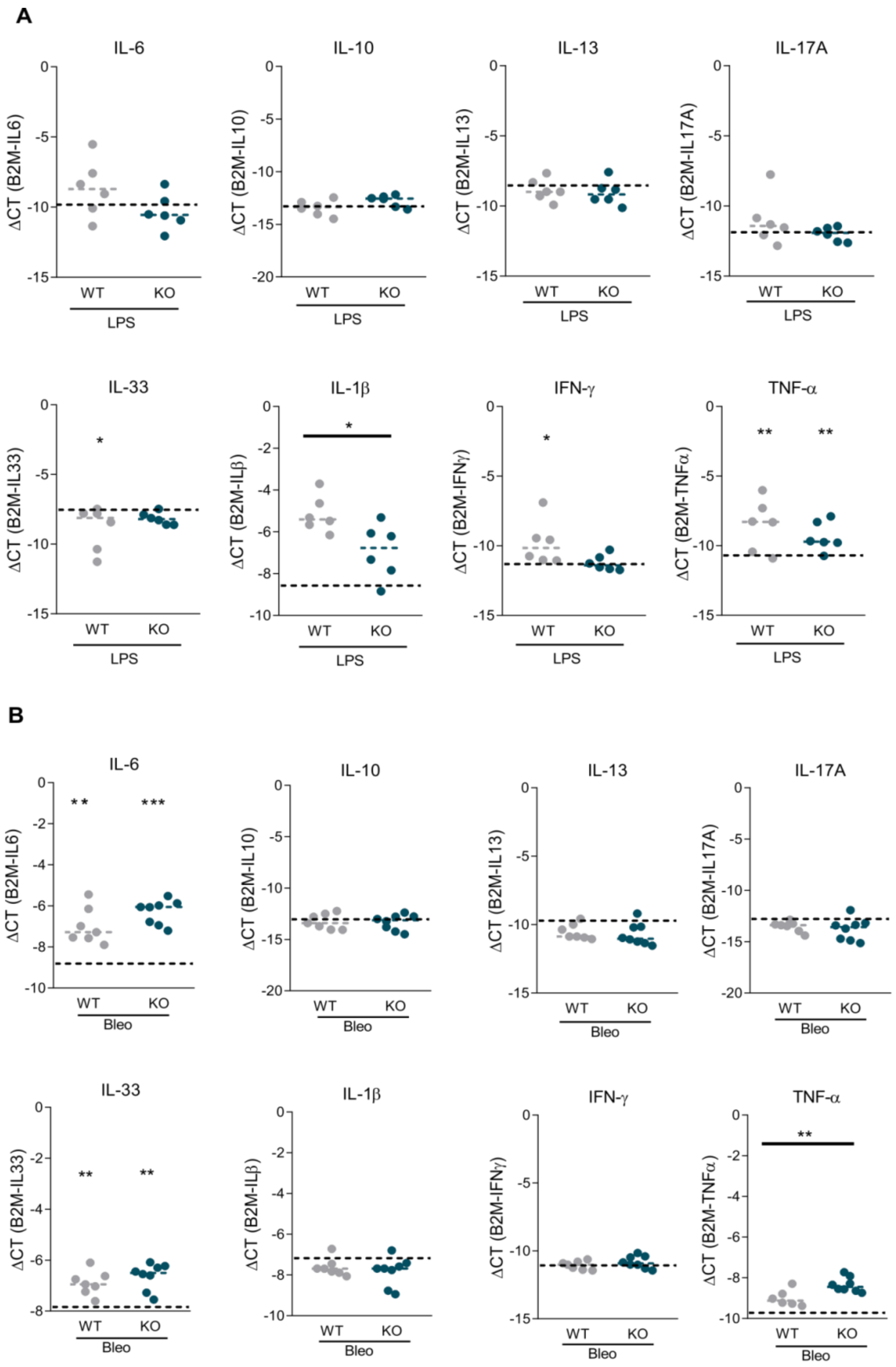

Supplementary Fig.S7 The inflammatory response in RGS5-/- mice in the acute lung injury models. (A) mRNA expression in the lungs of WT and RGS5-/- mice treated with saline and LPS for 24 hours (n ≥ 5). (B) mRNA expression in the lungs of WT and RGS5-/- mice upon saline and bleomycin treatment at day3 (n ≥ 7). ΔCT values, calculated by ΔCT = CT (reference gene) – CT (target gene). Dotted lines

represent mean of WT and RGS5-/- saline treated pooled together, as no differences were observed. Data are presented as scatter dot plots and analyzed using unpaired two-tailed t-test, * $p < 0.05$, ** $p < 0.01$ *** $p < 0.001$.

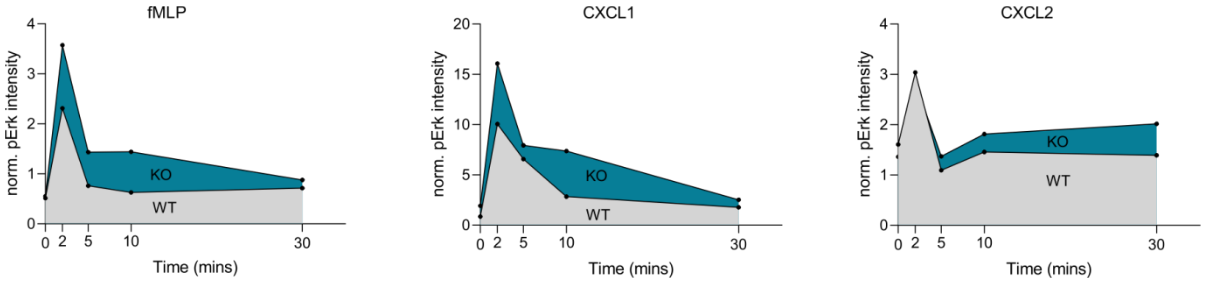

Supplementary Fig.S8 For quantification of the pERK (relative band intensities versus time), the area under the curve (AUC) was calculated. This representation of the data allows quantification of the pERK available throughout the detection period. Representative AUCs are shown for fMLP, CXCL1 and CXCL2 for both genotypes.
