# Supplementary material for: RGS5 Determines Neutrophil Migration in the Acute Inflammatory Phase of Bleomycin-Induced Lung Injury
Source: Int J Mol Sci. 2021 Aug 28;22(17):9342. doi: 10.3390/ijms22179342 (PMC8430858; doi:10.3390/ijms22179342)
Supplement: Supplementary file 1 [file ijms-22-09342-s001.zip › Supplementary Tables_final_PR.pdf]

Supplementary tables

Supplementary Table S1: Clinical characteristics of patients with interstitial lung disease (ILD) and controls with detailed use of material

Lung ID/Diagnosis	Age	Sex	BMI	FEV1(%)	FVC(%)	FEV1/ FVC(%)	LTOT	Material used
Control 1	43	Female	22.43	NA	NA	NA	no	Lung tissue, RNA
Control 2	40	Female	20.76	NA	NA	NA	no	Lung tissue, RNA
Control 3	17	Male	17.96	NA	NA	NA	no	Lung tissue, RNA
Control 4	62	Male	23.12	NA	NA	NA	no	Lung tissue, RNA
Control 5	59	Male	26.3	NA	NA	NA	no	Lung tissue, RNA
Control 6	70	Female	22.04	NA	NA	NA	no	Lung tissue, RNA
Control 7	58	Female	22.86	NA	NA	NA	no	Lung tissue, RNA
Control 8	42	Female	NA	NA	NA	NA	no	Lung tissue, RNA, protein
Control 9	24	Female	21.7	NA	NA	NA	no	Lung tissue, RNA
Control 10	71	Female	NA	NA	NA	NA	no	Lung tissue, RNA
Control 11	53	Female	31.11	NA	NA	NA	no	Protein
Control 12	39	Female	24.22	NA	NA	NA	no	Protein
Control 13	52	Male	NA	NA	NA	NA	no	Protein
Control 14	19	Male	NA	NA	NA	NA	no	Protein
Control 15	73	Female	NA	NA	NA	NA	no	Protein
ILD 1/HP	66.5	Female	26.93	49	47	104	yes	Lung tissue, RNA, protein
ILD 2/HP	64.2	Male	28.09	67	NA	NA	no	Lung tissue, RNA, protein
ILD 3/CTD	58.2	Female	17.31	30	31	97	yes	Lung tissue, RNA, protein
ILD 4/IPF	54	Male	24.15	40	34.9	115	yes	Lung tissue, protein
ILD 5/IPF	60	Male	21.88	34	36.2	94	yes	Lung tissue, RNA
ILD 6/IPF	72.3	Male	24.3	41	35	117	yes	Lung tissue, RNA, Protein
ILD 7/NSIP	48.4	Female	21.5	40	40	100	yes	Lung tissue, RNA, Protein
ILD 8/IPF	63.7	Male	23.03	47.5	40	119	yes	Lung tissue, RNA
ILD 9/HP	54.7	Male	32.05	31	27.5	113	yes	Lung tissue, RNA
ILD 10/IPF	58.2	Male	17.92	41	NA	NA	yes	Lung tissue, RNA
ILD 11/HP	68	Male	18.83	46.2	37.2	124	yes	Lung tissue, RNA
ILD 12/HP	67.5	Male	33.25	59.4	53.1	112	yes	Lung tissue
ILD 13/IPF	56	Male	NA	NA	NA	76	NA	Lung tissue
ILD 14/IPF	63	Male	NA	NA	NA	93	NA	Lung tissue
ILD 15/IPF	66	Female	NA	NA	NA	89	NA	Lung tissue

BMI: Body mass index (Kg/m^2); FEV1: Forced expiratory volume in 1 s; FVC: Forced vital capacity; LTOT: Long-term oxygen therapy; CTD: Connective tissue diseases; HP: Hypersensitivity pneumonitis; IPF: Idiopathic pulmonary fibrosis; NSIP: Nonspecific interstitial pneumonia; NA: Not available.

Supplementary Table S2: Clinical characteristics of patients with acute respiratory distress syndrome (ARDS) with detailed use of material

Lung ID	Age	Sex	Background	Modified APACHE II	PaO_2/FiO_2 (mmHg)	Material used
ARDS 1	40	Female	Pneumonia	11	83	Lung tissue
ARDS 2	51	Female	Trauma	6	181.5	Lung tissue
ARDS 3	67	Male	Sepsis	20	109	Lung tissue

Supplementary Table S3: Antibodies for flow-cytometry analysis of immune cells from BALF and lung tissue

Antigen	Label	company	Clone	isotype	dilution
CD45	PerCP-Cy5.5	eBioscience	30-F11	Rat IgG2b. κ	1:200
CD45	FITC	Thermo Fisher	30-F11	Rat IgG2b. κ	1:200
CD11b	ef506	Thermo Fisher	M1/70	Rat IgG2b. κ	1:50
CD11c	ef450	Thermo Fisher	N418	Hamster IgG	1:50
Gr-1(Ly6G/Ly6C)	PE-Cy7	Biolegend	RB6-8C5	Rat IgG2b. κ	1:800
CD64	AF647	BD Bioscience	X54-5/7.1	Mouse IgG1. κ	1:20
Siglec-F	PE	BD Bioscience	E50-2240	Rat IgG2a. κ	1:20
MHC-II	APC-Cy7	Biolegend	M5/114.15.2	Rat IgG2b. κ	1:400
CD3	AF700	Thermo Fisher	eBio500A2	Hamster IgG	1:50
CD4	APC	Biolegend	GK 1.5	Rat IgG2b. κ	1:100
CD8	PE	Biolegend	53-6.7	Rat IgG2a. κ	1:100
CD19	BB515	BD Bioscience	1D3	Rat IgG2a. κ	1:50
NK1.1	SB600	eBioscience	PK136	Mouse / IgG2a. κ	1:20
γδTCR	ef450	Thermofischer	eBiogL3	Hamster IgG	1:50

Supplementary Table S4: Antibodies for western blots and stainings

Antibodies	Company/cataloge number	Experiments	Sample/Dilution
RGS5	Santa cruz/(B-4) sc514184	IHC (mouse and human)	Lung tissue/1:250
		western blot	Protein/1:1000
	Abcam/196799	IHC	Neutrophils/1:100
Ly6G	Biolegend/12706	IHC	Lung tissue/ 1:200
	Biolegend/127618	FACS	Bone marrow. blood.spleen/1:800
		FACS	Neutrophils/1:800
Cd11b	Invitrogen/69011282	FACS	Neutrophils/1:50
CXCR2	Biolegend/149312	FACS	Neutrophils/1:40
CXCR4	Biolegend/146505	FACS	Neutrophils/1:40
Erk	Cell signaling/9102S	Western blot	Protein/ 1:1000
pErk	Cell signaling/9101S	Western blot	Protein/ 1:1000
GAPDH	Santa cruz/(FL-335) sc25778	Western blot	Protein/ 1:1000
ß-actin	Santa cruz/(C-4) sc47778	Western blot	Protein/ 1:1000

Supplementary Table S5: Primers sequences for human and mouse

Primers	Species	Forward (5'-3')	Reverse (5'-3')
RGS1	Human	TTGAGTTCTGGCTGGCTTGTG	GCAGCATCTGAATGCACAAATG
RGS2	Human	TTCTGGCTGGCCTGTGAAG	GCAGTTGTAAAGCAGCCACTTG
RGS3	Human	GCACACCAAGGACAACCTGC	ACGGAGAAAGCGAGGGTACG
RGS4	Human	ACCAGGGAAGAGACAAGCCG	ACTTGAGGAAGCGGCGGTAG
RGS5	Human	ACCTGGTGGAACCTTCCCTG	AACTCAGAGCGCACAAAGCG
RGS8	Human	CTGCATTCCGTGCCTTCTTG	TTTACCTCCCGTGGAGCCTG
RGS13	Human	CGGTGGAGCAGAATTTCTAGGG	TGTTTCAGTGGGTTCCTGAATG
RGS16	Human	AACCTGCAGACTGCCACAGC	CGGTAAGCAGGCGACTTCAG
RGS18	Human	CAAGGGACCTCAACAAATTCACC	TGGAGGGTAGGTTGAGTGATGC
β2M	Human	CCTGGAGGCTATCCAGCGTACTCC	TGTCGGATGGATGAAACCCAGACA
CXCL1	Mouse	CCTTGACCCTGAAGCTCCCT	CGGTGCCATCAGAGCAGTCT
CXCL2	Mouse	ACATCCAGAGCTTGAGTGTGA	TTCAGGGTCAAGGCAAACTT
β2M	Mouse	CGGCCTGTATGCTATCCAGAAAACC	TGTGAGGCGGGTGGAACTGTG
IL-1β	Mouse	GCCACCTTTTGACAGTGATGAG	GACAGCCCAGGTCAAAGGTT
IL-6	Mouse	ACAACCACGGCCTTCCCTACTT	CACGATTTCCCAGAGAACATGTG
IL-10	Mouse	ATAACTGCACCCACTTCCCA	TCATTTCCGATAAGGCTTGG
IL-13	Mouse	GCCAAGATCTGTGTCTCTCCC	CCAGGTCCACACTCCATACC
IL-17	Mouse	AGGACGCGCAAACATGAGTC	GGACACGCTGAGCTTTGAGG
IL-33	Mouse	ACCATGAGACCTAGAATGAAGTAT	TTAGATTTTCGAGAGCTTAAAC
IFN-γ	Mouse	CAGCAACAGCAAGGCGAAAAAGG	TTTCCGCTTCCTGAGGCTGGAT
TNF-α	Mouse	CATCTTCTCAAAATTCGAGTGACAA	TGGGAGTAGACAAGGTACAACCC
