# Supplementary figures and images for: RGS5 Determines Neutrophil Migration in the Acute Inflammatory Phase of Bleomycin-Induced Lung Injury
Source: Int J Mol Sci. 2021 Aug 28;22(17):9342. doi: 10.3390/ijms22179342 (PMC8430858; doi:10.3390/ijms22179342)

**Figure 1C**

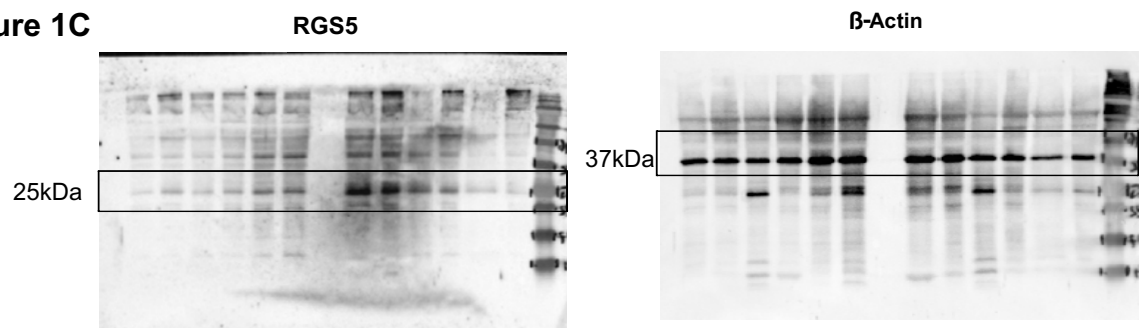

**Figure 2C**

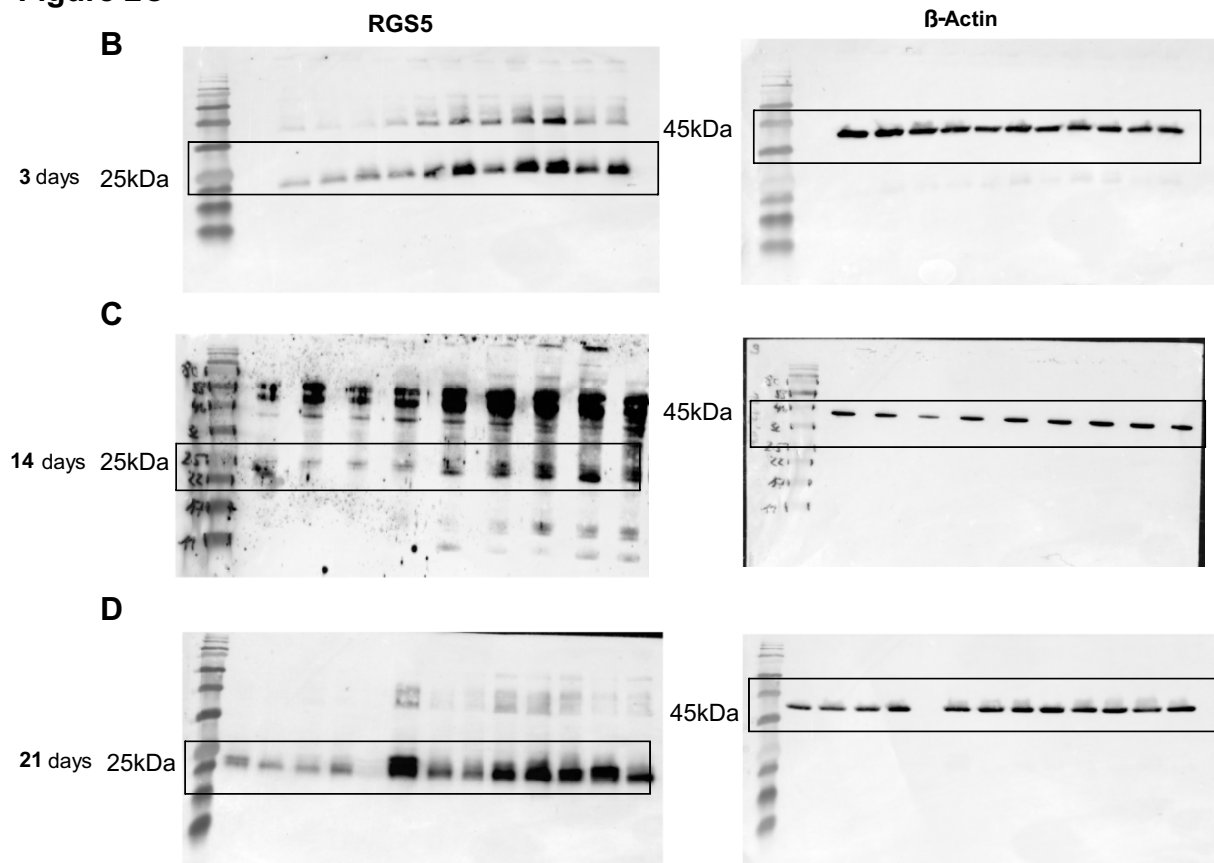

fMLP

Figure 7A

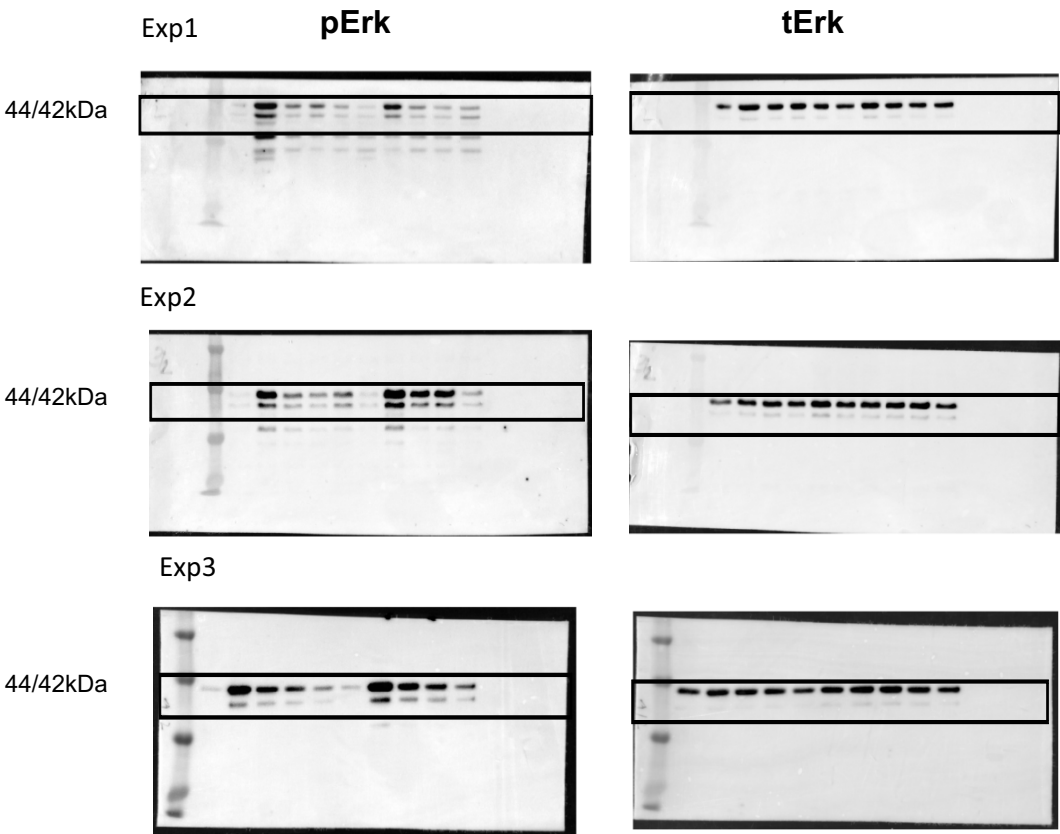

Figure 7A

CXCL1

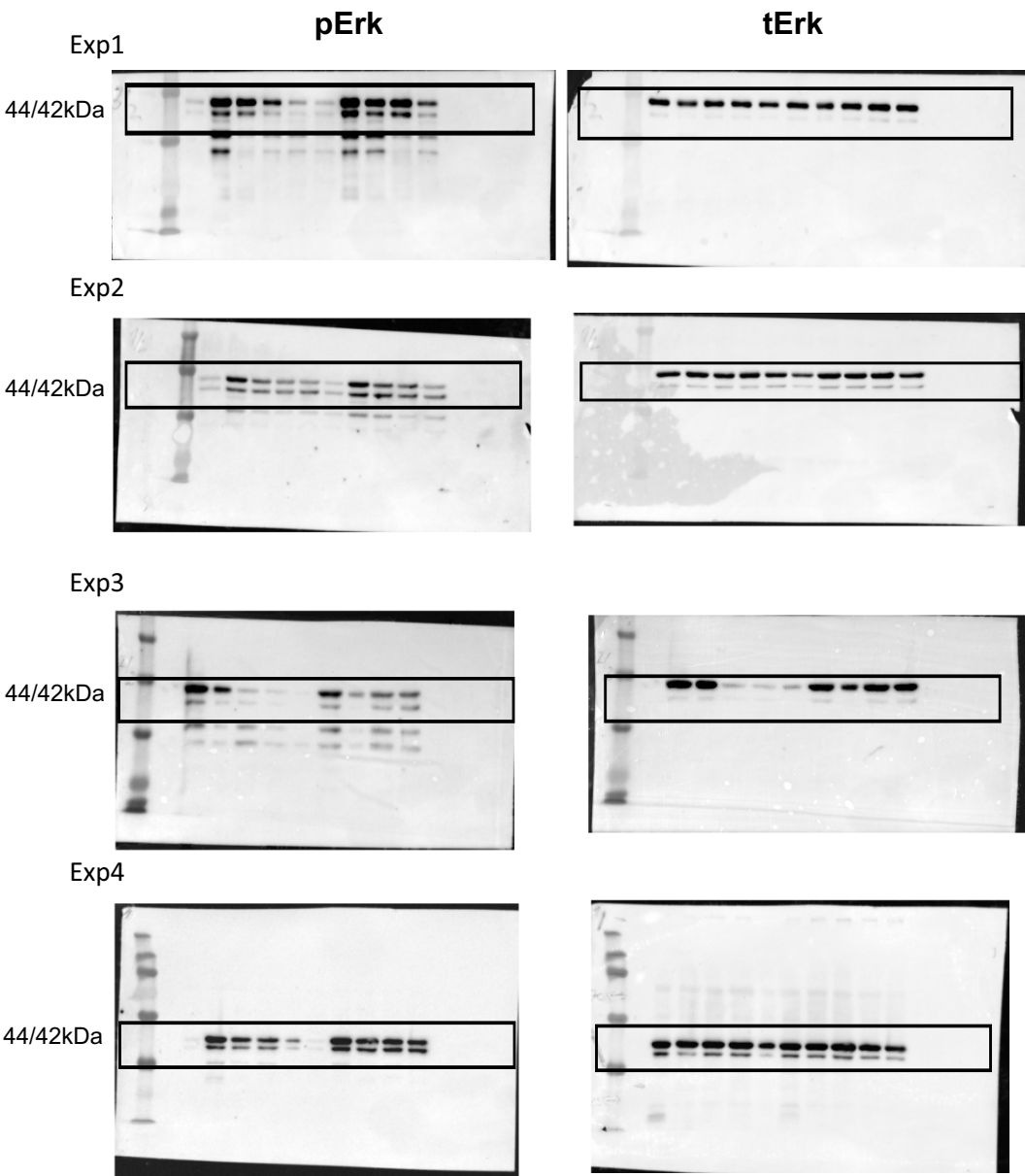

**Figure 7A**

**CXCL2**

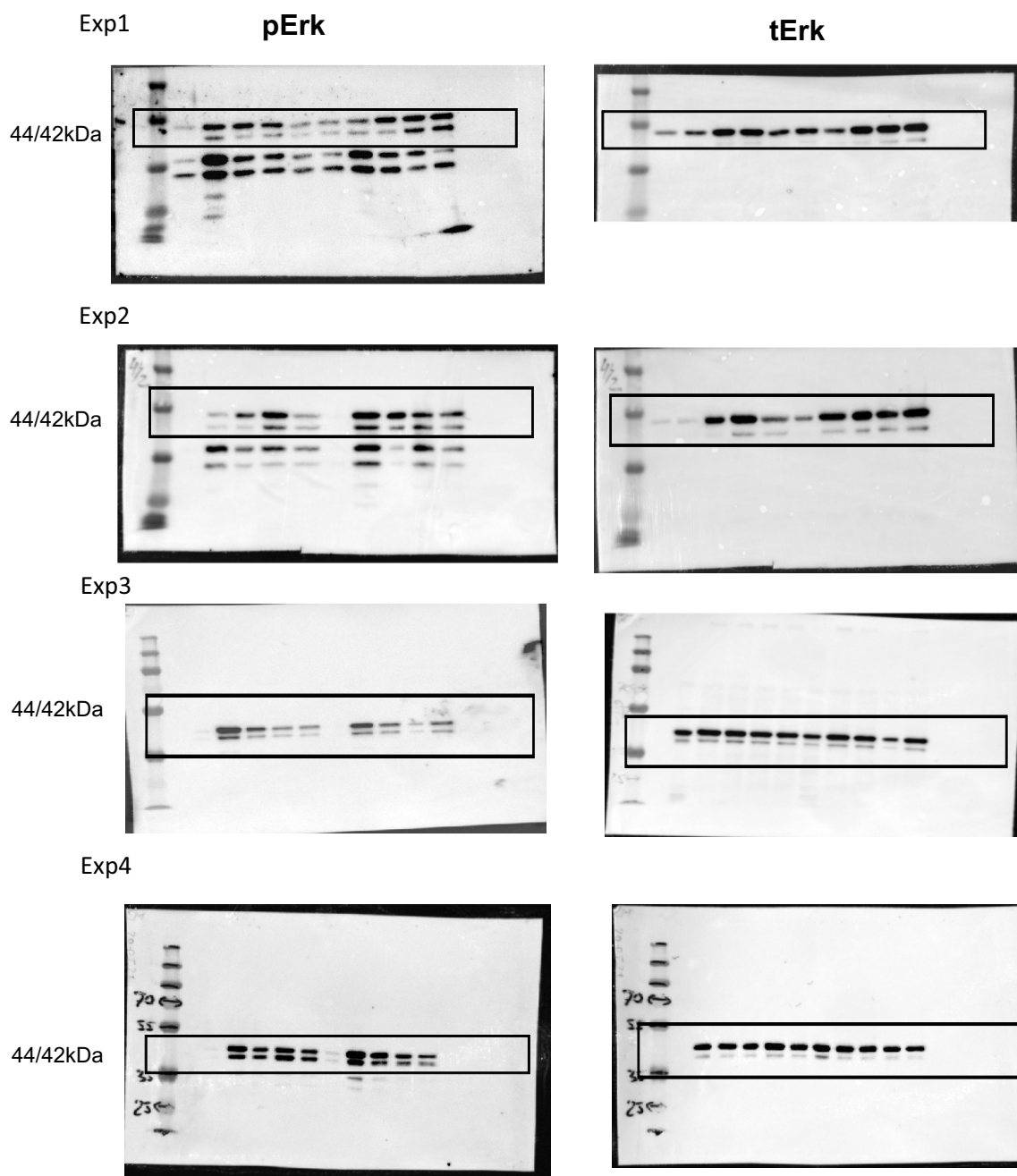

Supplement: Supplementary file 1 [file ijms-22-09342-s001.zip › westernblot_uncropped_revised_PR.pdf]
